# Supplementary material for: HSF1 is a driver of leukemia stem cell self-renewal in acute myeloid leukemia
Source: Nat Commun. 2022 Oct 16;13:6107. doi: 10.1038/s41467-022-33861-1 (PMC9573868; doi:10.1038/s41467-022-33861-1)
Supplement: Supplementary file 1 — Supplementary Information [file 41467_2022_33861_MOESM1_ESM.pdf]

HSF1 is a Driver of Leukemia Stem Cell Self-Renewal in Acute Myeloid Leukemia

Supplementary Information

SUPPLEMENTARY FIGURES

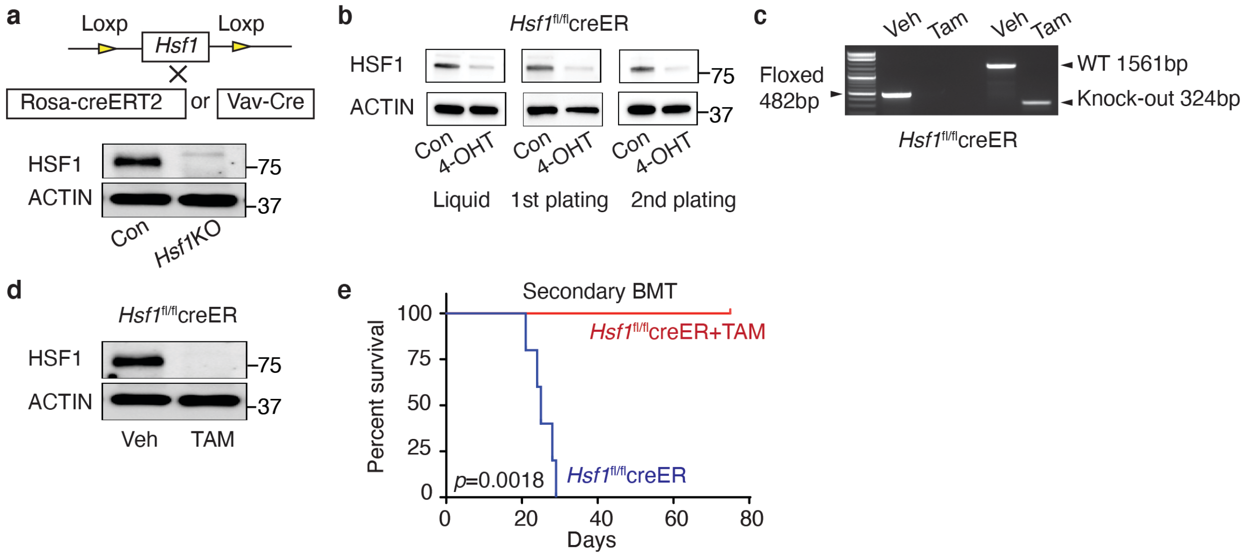

Figure S1: HSF1 is required for the initiation and maintenance of MLL-AF9–induced AML. **a** Scheme of experimental design (upper) and confirmation of HSF1 protein deletion in whole BM (lower). Protein was extracted from 6-week-old Control (Con) or *Hsf1*KO mice (n=4 independent replicates). **b** HSF1 protein expression in MLL-AF9 leukemia cells in the presence or absence of 4-OHT. Left panel, samples prepared from liquid culture; middle panel, samples from primary methycellulose plating; right panel, samples from secondary methycellulose plating in the absence of further Tam treatment. Actin was used as a loading control (n=2 independent replicates). **c** Genomic *Hsf1* deletion in Tam- or vehicle (Veh, corn oil + ethanol)-treated MLL-AF9 *Hsf1*$^{fl/fl}$creER leukemia cells. DNA was extracted from Tam-or vehicle-treated leukemia cells and amplified by PCR (n=4 independent replicates). **d** HSF1 protein in vehicle- or Tam-treated *Hsf1*$^{fl/fl}$creER leukemia cells (n=2 independent replicates). Actin was used as a loading control. **e** Survival curve of mice receiving whole BM cells from primary recipient mice receiving full-blown MLL-AF9 *Hsf1*$^{fl/fl}$creER leukemic mice treated with two cycles of Tam

(from **Fig. 1l)** (n=5 mice/group). Log-rank test, **p*=0.0018. Source data are provided as a Source Data file.

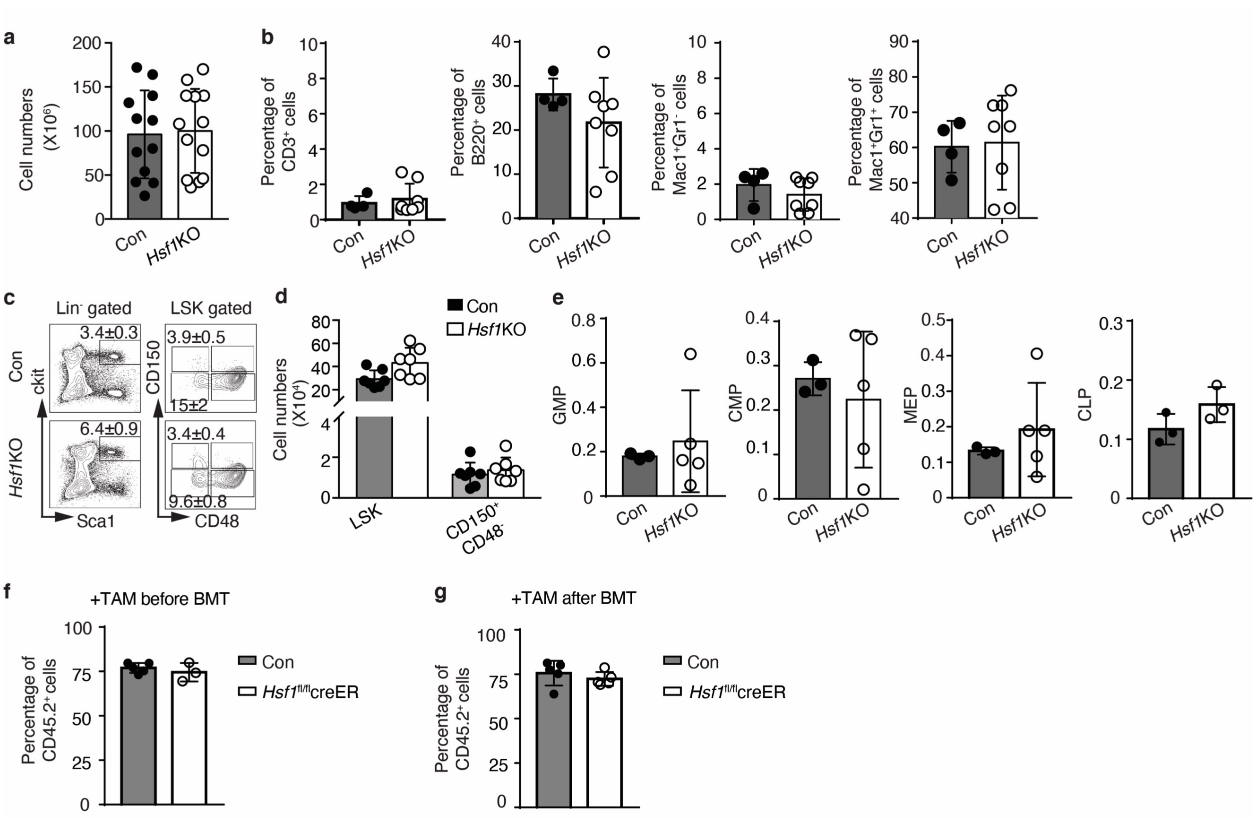

Figure S2: HSF1 is dispensable for steady-state hematopoiesis. **a** Whole BM cell counts of Control or *Hsf1*KO mice (Con n=12 mice; *Hsf1*KO n=13 mice). **b** Frequencies of $CD3^+$ T cells, $B220^+$ B cells, $Mac1^+Gr1^-$ monocytes and $Mac1^+Gr1^+$ granulocytes in Control (n=4 mice) or *Hsf1*KO mice (n=8 mice). **c, d** Frequencies (**c**) and absolute numbers (**d**) of LSK ($lin^-cKit^+Sca1^+$) and HSCs ($CD150^+CD48^-LSK$) of Control or *Hsf1*KO mice (n=7 mice/group). **e** Frequencies of GMP (granulocyte-macrophage progenitor, $Lin^-cKit^+Sca1^-CD34^+CD16/32^+$), CMP (common myeloid progenitor, $Lin^-cKit^+Sca1^-CD34^+CD16/32^-$), MEP (megakaryocyte-erythrocyte progenitor, $Lin^-cKit^+Sca1^-CD34^-CD16/32^-$) and CLP (common lymphoid progenitor, $Lin^-cKit^{low}Sca1^{low}CD127^+$) of Control (n=3 mice) or *Hsf1*KO mice (n=5 mice). **f** Donor chimerisms of mice transplanted with $2x10^6$ whole BM cells from vehicle (Con, n=5 mice) or Tam treated *Hsf1*$^{fl/fl}$creERT mice (n=3 mice). **g** Donor chimerisms of mice transplanted with $2x10^6$ whole BM

cells from *Hsf1*$^{fl/fl}$creERT mice, followed by vehicle or TAM treatment 8 weeks post transplantation (n=5 mice/each group). Donor chimerisms were determined two weeks after the last dose of Tam. In **a, b, d-g**, Data are presented as mean values +/- SEM. Source data are provided as a Source Data file.

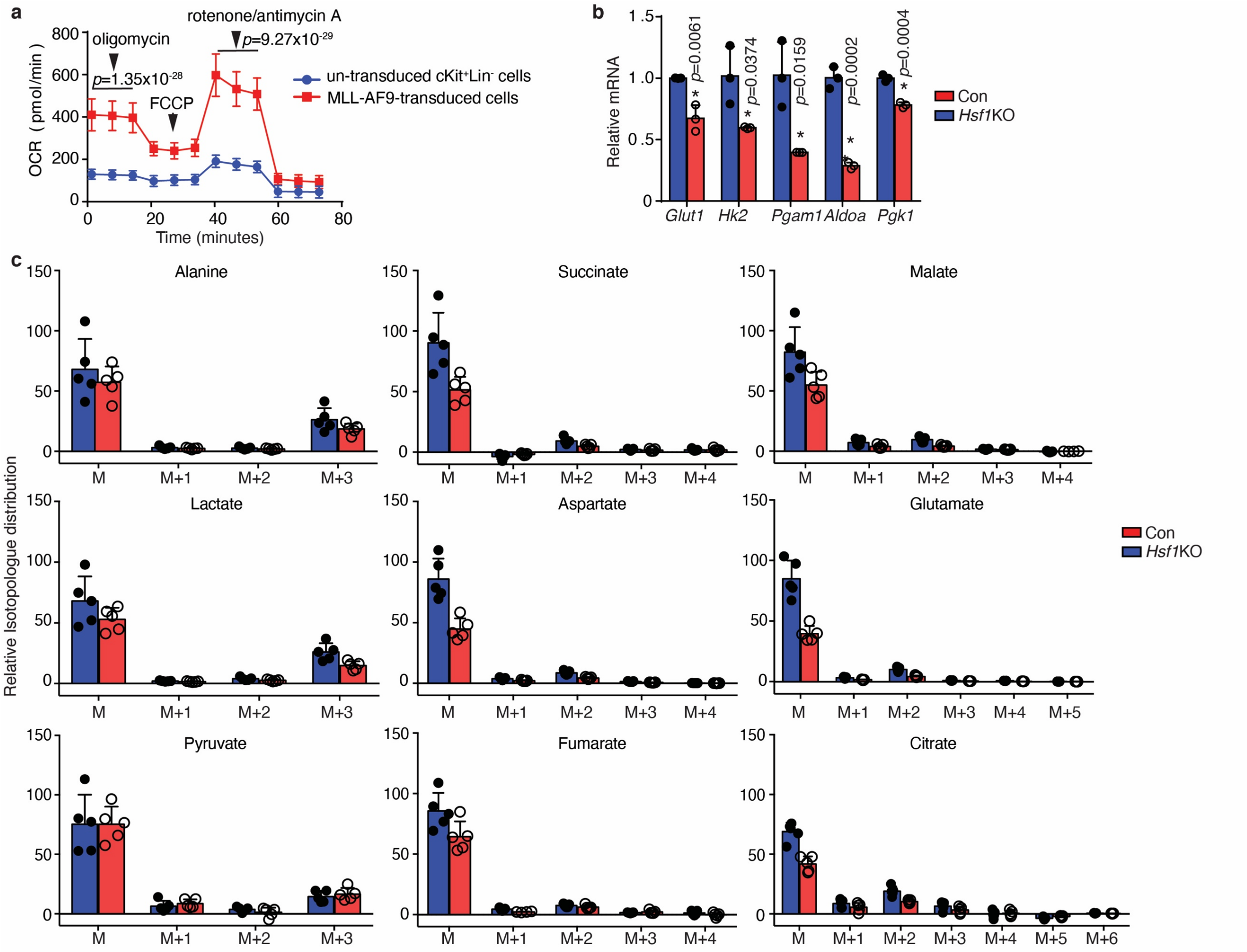

Figure S3: **HSF1 is a key regulator of oxidative phosphorylation in AML. a** OCR in MLL-AF9 transduced or non-tranduced BM cKit+Lin- cells (n=10 independent replicates). **b** Relative expression of glycolysis-related genes by qPCR in control or *Hsf1* deleted (*Hsf1*KO) mouse MLL-AF9 LSCs (n=3 independent replicates). **c** Labelling fraction of each isotopologue for the selected metabolites (n=5 independent replicates). In **a-c**, two-tailed t test was used. Data are presented as mean values +/- SEM. Source data are provided as a Source Data file.

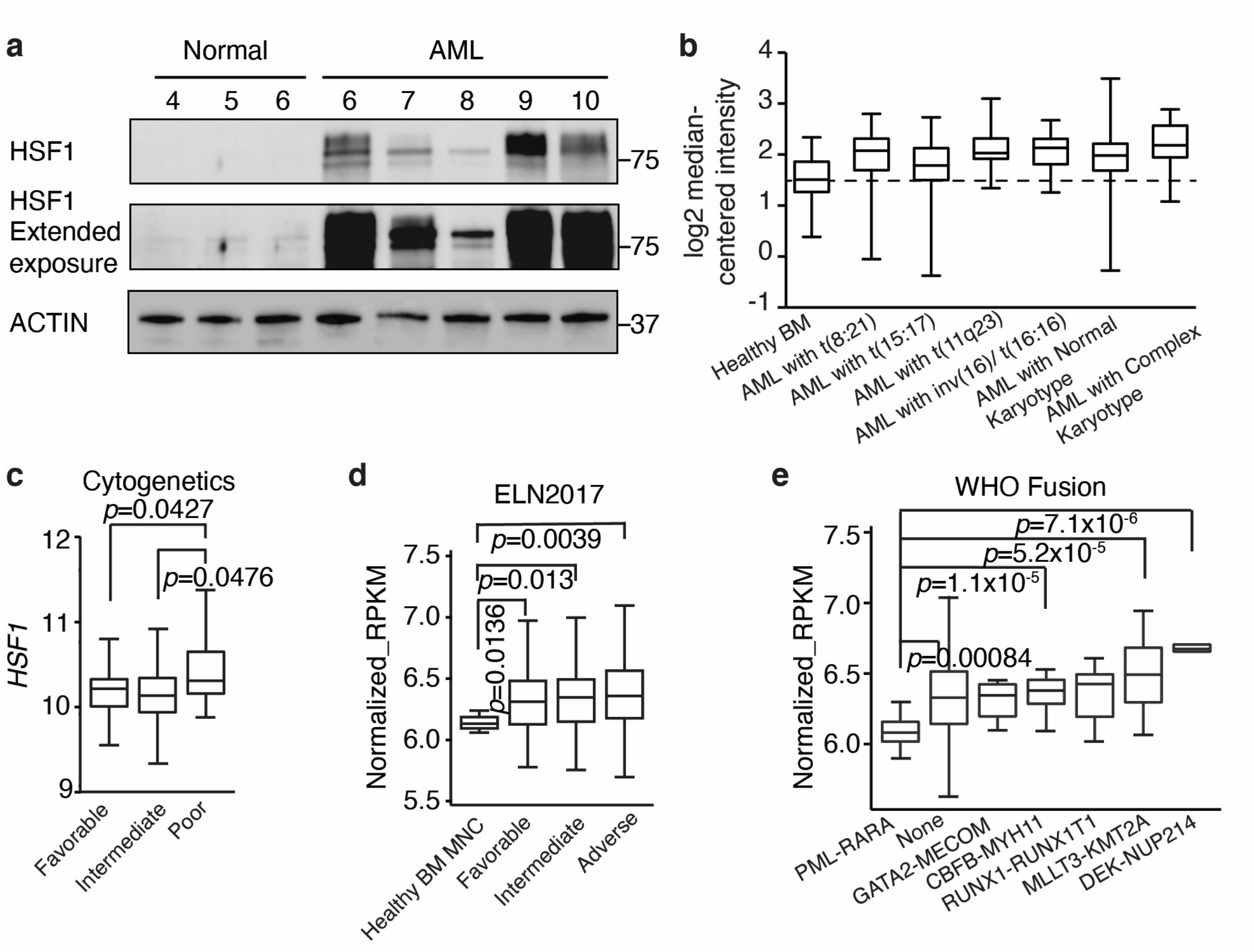

Figure S4: HSF1 is not required for human HSPC repopulation but is critical for maintenance of LSC self-renewal. a Expression of HSF1 protein in 3 normal BM samples and 5 primary AML BM samples. Actin is used as a loading control. **b-e** Expression of *HSF1* in different subtypes of AML. Data are from Oncomine (**b**, Haferlach leukemia; no significant differences among AML categories, overall higher than healthy controls), TCGA (**c**) and Tyner et al.[55] (**d, e**). In **b-e**, Boxes display median expression values and contain data from the 25th to 75th percentiles with the bars representing the 10th and 90th percentiles, respectively. In (**b**), the patient numbers in each category are: healthy BM 74, AML with t(8;21) 40, t(15;17) 37,

t(11q23) 38, inv(16)/t(16;16) 28, AML with normal karyotype 351, and AML with complex karyotype 48. In **c-e**, two-sided t-test was used to compared indicated two groups.

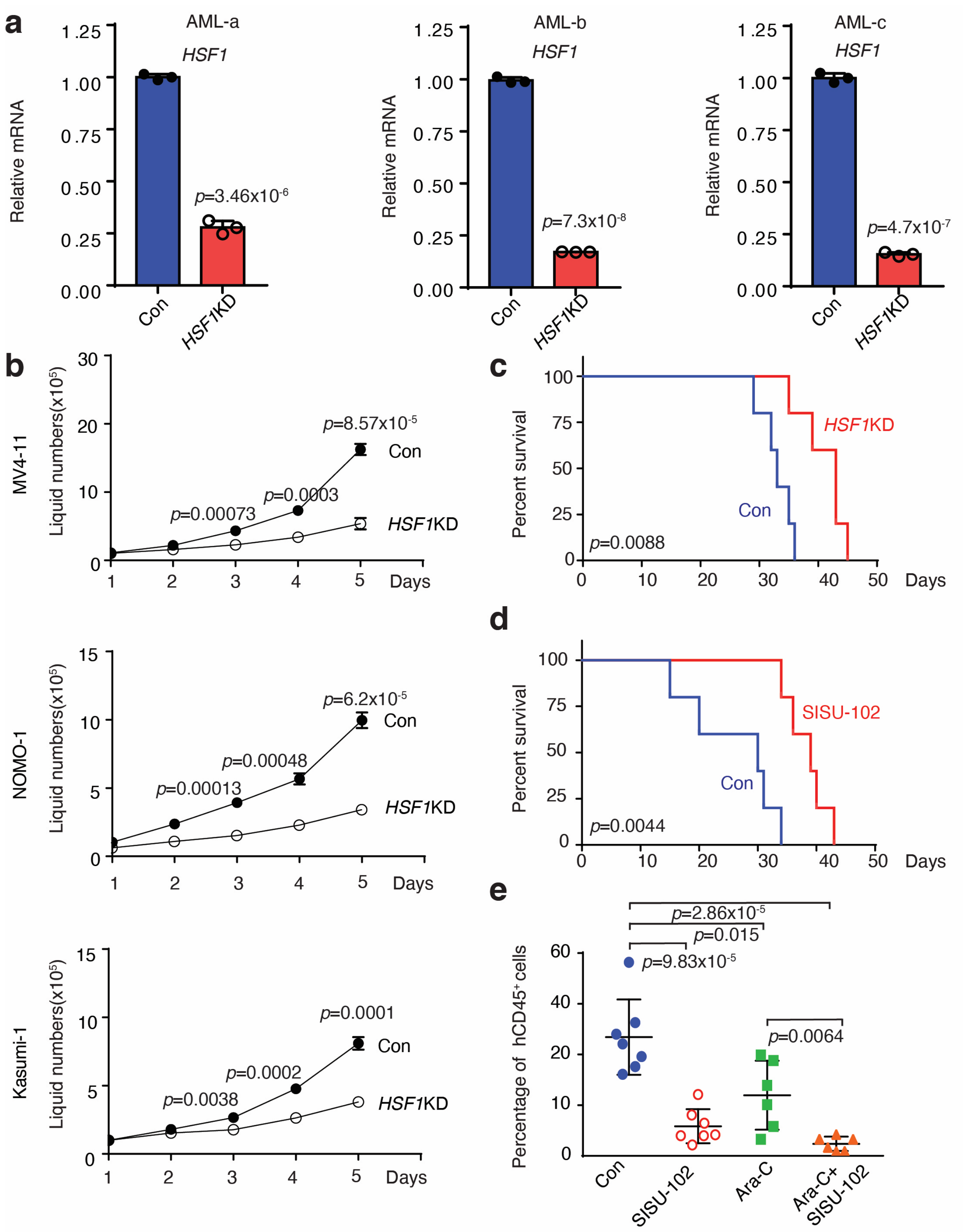
a
AML-a
HSF1
Relative mRNA
1.25
1.00
0.75
0.50
0.25
0.00
p=3.46x10-6
Con
HSF1KD
AML-b
HSF1
Relative mRNA
p=7.3x10-8
Con
HSF1KD
AML-c
HSF1
Relative mRNA
p=4.7x10-7
Con
HSF1KD
b
MV4-11
Liquid numbers(x10⁵)
p=8.57x10-5
Con
p=0.0003
p=0.00073
HSF1KD
Days
NOMO-1
Liquid numbers(x10⁵)
p=6.2x10-5
Con
p=0.00048
p=0.00013
HSF1KD
Days
Kasumi-1
Liquid numbers(x10⁵)
p=0.0001
Con
p=0.0002
p=0.0038
HSF1KD
Days
c
Percent survival
HSF1KD
Con
p=0.0088
Days
d
Percent survival
SISU-102
Con
p=0.0044
Days
e
Percentage of hCD45+ cells
p=2.86x10-5
p=0.015
p=9.83x10-5
p=0.0064
Con
SISU-102
Ara-C
Ara-C+
SISU-102

Figure S5: HSF1 is critical for the maintenance of LSC self-renewal. a Relative expression of *HSF1* mRNA in Cas9 (Con)- or CRISPR-Cas9-mediated *HSF1* knockdown (HSF1KD) in 3 primary human AMLs used for mouse transplantation (see **Fig. 6g**). **b** Growth of human AML cell line MV4-11, NOMO-1, or Kasumi-1 with or without CRISPR-Cas9-mediated *HSF1* knockdown (n=3 independent replicates each). **c** Survival curve of mice receiving primary human AML cells with or without CRISPR-Cas9-mediated *HSF1* knockdown (n=5 mice/group). **d** Survival curve of mice receiving primary human AML cells treated with or without HSF1 small molecular inhibitor SISU-102 (n=5 mice/group). In **c** and **d**, log-rank test was used. **e** Engraftment of primary human AML cells and treated (2 weeks after transplantation) with vehicle (n=7 mice), SISU-102 (5 mg/kg, n=7 mice), Ara-C (30 mg/kg, n=6 mice) or SISU-102 (5 mg/kg) + Ara-C (30 mg/kg) (n=6 mice). In **a, b, e**, two-tailed t test was used, and data are presented as mean values +/- SEM. Source data are provided as a Source Data file.

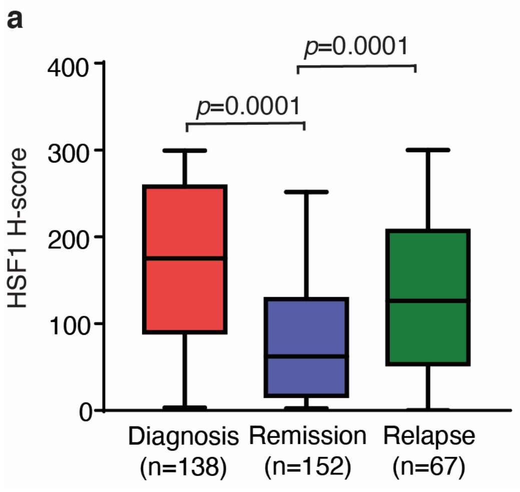

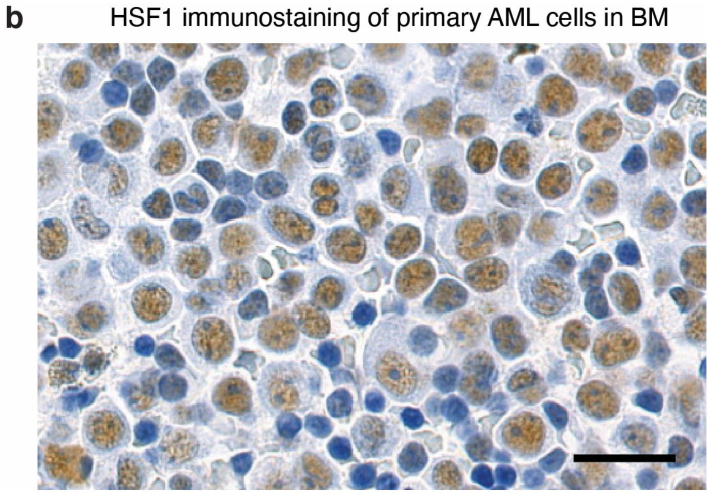

Figure S6: Expression of HSF1 may serve as a marker to monitor malignant status. a HSF1 H-score index in all AML samples at diagnosis, remission, or relapse. ANOVA with LSD post-hoc analysis was used. Box plots show median and first/third quartiles. Total number of AML patients was 162. Among these 162 patients, 138 samples at diagnosis (some were diagnosed at other hospitals), 152 samples at remission, and 67 samples at relapse were available for HSF1 immunostaining and scanning for analysis. The number of the same patients with samples at diagnosis, remission, and relapse, is 39, and the data presented as Fig. 6**o**. **b** Representative high-resolution image of HSF1 immunohistochemical staining (brown, nuclear) in AML BMs (n=162 primary AML samples). Scale bar, 20 μM. Note the nuclear staining of HSF1 protein, which is used for determination of the H-score index. Source data are provided as a Source Data file.

Supplementary Table

Human primary AML samples used for xenograft transplantation

AML-a	AML with normal Karyotype and DNMT3a and TET2 mutations
AML-b	t-AML with FLT3/MLL3 missense mutation and t(9;11)(p22;q23)
AML-c	AML with normal karyotype and FLT3 ITD/NPM1 mutations
AML-d	AML with t(6; 9)(p22; q34) and FLT3 ITD mutation
AML-e	AML with complex karyotype (t-AML) and TP53 mutation
AML-f	AML with normal karyotype and NPM1, IDH1 and PTPN11 mutations
