# Supplementary material for: HSF1 is a driver of leukemia stem cell self-renewal in acute myeloid leukemia
Source: Nat Commun. 2022 Oct 16;13:6107. doi: 10.1038/s41467-022-33861-1 (PMC9573868; doi:10.1038/s41467-022-33861-1)
Supplement: Supplementary file 3 — Reporting Summary [file 41467_2022_33861_MOESM3_ESM.pdf]

nature portfolio

Corresponding author(s): Chen Zhao, MD. PhD

Last updated by author(s): Sep 11, 2022

Reporting Summary

Nature Portfolio wishes to improve the reproducibility of the work that we publish. This form provides structure for consistency and transparency in reporting. For further information on Nature Portfolio policies, see our Editorial Policies and the Editorial Policy Checklist.

Statistics

For all statistical analyses, confirm that the following items are present in the figure legend, table legend, main text, or Methods section.

n/a	Confirmed	
☐	☒	The exact sample size (*n*) for each experimental group/condition, given as a discrete number and unit of measurement
☐	☒	A statement on whether measurements were taken from distinct samples or whether the same sample was measured repeatedly
☐	☒	The statistical test(s) used AND whether they are one- or two-sided<br>*Only common tests should be described solely by name; describe more complex techniques in the Methods section.*
☒	☐	A description of all covariates tested
☒	☐	A description of any assumptions or corrections, such as tests of normality and adjustment for multiple comparisons
☐	☒	A full description of the statistical parameters including central tendency (e.g. means) or other basic estimates (e.g. regression coefficient) AND variation (e.g. standard deviation) or associated estimates of uncertainty (e.g. confidence intervals)
☐	☒	For null hypothesis testing, the test statistic (e.g. F, t, r) with confidence intervals, effect sizes, degrees of freedom and P value noted<br>*Give P values as exact values whenever suitable.*
☒	☐	For Bayesian analysis, information on the choice of priors and Markov chain Monte Carlo settings
☒	☐	For hierarchical and complex designs, identification of the appropriate level for tests and full reporting of outcomes
☒	☐	Estimates of effect sizes (e.g. Cohen's d, Pearson's r), indicating how they were calculated

Our web collection on statistics for biologists contains articles on many of the points above.

Software and code

Policy information about availability of computer code

Data collection	Western blot data were collected by ChemiDoc Touch Imaging system (Bio-Rad)<br>Real-time PCR data were collected by QuantStudio 3 Real-Time PCR System<br>Flow cytometry data were collected by LSRII and BD Aria.<br>For mass spectrometric experiments, metabolites were detected using a Thermo ISQ 7000 mass spectrometer.<br>RNA-seq Libraries were sequenced on Illumina's HiSeq2000<br>OCR, ECAR and ATP production data were collected by the Agilent Seahorse XFe96 Analyzer.<br>Illumina CASAVA 1.8.2 was used to generated sequence data in fastq format.<br>DESeq2 v1.30.1 R package 81 was used for differential expression analysis .<br>Gene set enrichment analysis (GSEA) 3.0 (http://software.broadinstitute.org82) was used to identify statistically enriched gene sets.
Data analysis	Software Flowjo version 10 were used for FACS analyses.<br>Prism software (GraphPad, version 7) was used for statistical analyses and to draw graphs in the study.<br>The Case Viewer digital image analysis software version 2.4 was used for quantification of immunoblot intensity.<br>Adobe Photoshop CC and Illustrator CC was used to crop images from unprocessed images.<br>Acquired GC-MS data were processed by Thermo Scientific TraceFinder 4.1 software. MetaboAnalyst 4.0 was used for further statistical processing and visualization

For manuscripts utilizing custom algorithms or software that are central to the research but not yet described in published literature, software must be made available to editors and reviewers. We strongly encourage code deposition in a community repository (e.g. GitHub). See the Nature Portfolio guidelines for submitting code & software for further information.

Data

Policy information about availability of data

All manuscripts must include a data availability statement. This statement should provide the following information, where applicable:

- Accession codes, unique identifiers, or web links for publicly available datasets
- A description of any restrictions on data availability
- For clinical datasets or third party data, please ensure that the statement adheres to our policy

The RNA-seq and CUT&RUN data reported in this paper have been deposited in the National Center for Biotechnology Information's Sequence Read Archive (SRA) database with the BioProject ID: PRJNA782637 (https://www.ncbi.nlm.nih.gov/Traces/study/?acc=PRJNA782637&o=acc_s%3Aa). All data are included in the Supplemental Information or available from the authors upon reasonable requests. The raw numbers for charts and graphs are available in the Source Data file whenever possible. Source data are provided with this paper

Field-specific reporting

Please select the one below that is the best fit for your research. If you are not sure, read the appropriate sections before making your selection.

☒ Life sciences ☐ Behavioural & social sciences ☐ Ecological, evolutionary & environmental sciences

For a reference copy of the document with all sections, see nature.com/documents/nr-reporting-summary-flat.pdf

Life sciences study design

All studies must disclose on these points even when the disclosure is negative.

Sample size	Since this is a preclinical study, no sample size calculation was performed for all the experiments.<br>For cell based experiments, samples are triplicated and more than three independent experiments were performed.<br>For mice experiments, sample size for biological replicates was always more or equal 3.<br>Detailed sample sizes are stated in figure legends or source data files.
Data exclusions	No data were excluded from the analyses.
Replication	Experiments were performed in three independent biological replicates. All attempts were successful at replication although there were some variations of values. De-identified human experiments were not replicated, unique cases were collected.
Randomization	Simple randomization was performed to assign mice, or cell lines or patient samples were cultured in the same conditions and randomly divided into different treatment/conditions for experiments.
Blinding	For immunohistochemistry, RNA-seq and CUT&RUN, the investigators used assigned sample ID during data collection and data analysis, and were not given grouping allocation information.<br>Data analysis was performed and confirmed by multiple investigators.

Reporting for specific materials, systems and methods

We require information from authors about some types of materials, experimental systems and methods used in many studies. Here, indicate whether each material, system or method listed is relevant to your study. If you are not sure if a list item applies to your research, read the appropriate section before selecting a response.

Materials & experimental systems

n/a	Involved in the study	
☐	☒	Antibodies
☐	☒	Eukaryotic cell lines
☒	☐	Palaeontology and archaeology
☐	☒	Animals and other organisms
☐	☒	Human research participants
☒	☐	Clinical data
☒	☐	Dual use research of concern

Methods

n/a	Involved in the study	
☒	☐	ChIP-seq
☐	☒	Flow cytometry
☒	☐	MRI-based neuroimaging

Antibodies

Antibodies used	antibodies used for flow cytometry analysis and sorting were purchased from eBioscience/Thermo Fisher Scientific or BD Biosciences.<br>Antibodies (in the order of name, catalog number, and clone name) from eBioscience/Thermo Fisher Scientific:

	CD3e-PE, 12-0031-83, 145-2C11; CD4-PE, 12-0042-83, RM4-5; CD5-PE, 12-0051-82, 53-7.3; CD8-PE, 12-0081-83, 53-6.7; B220-PE, 12-0452-83, RA3-6B2; CD11b-PE, 12-0112-83, M1/70; CD16/CD32 -PECy7, 25-0161-82, 93;CD16/CD32-APC, 17-0161-82, 93;CD16/32-PE, 12-0161-82, 93;Gr1-PE, 12-5931-83, RB6-8C5; Ter119-PE, 12-5921-83;CD127-PE, 12-1271-82, A7R34; Sca1-Percp-Cy5.5, 45-5981-82, D7; Scar1-PE, 12-5981-83, D7Scar1-PECy5, 25-5981-82, D7; Sca-1 Percp-Cy5.5, 45-5981-82, D7;CD11b-FITC, 11-0112-82, M1/70; Gr1-FITC, 11-5931-82, RB6-8C5; CD127-FITC, 12-1271-81, A7R34; CD34-FITC, 11-0341-85, RAM34; CD45.1-APC, 17-0453-82, A20; CD48-APC, 17-0481-82, HM48-1; CD48-PE, 12-0481-82, HM48-1;CD45.2-PECy7, 25-0454-82, 104; CD150-PECy7, 25-1502-82, mShad150; CD135-APC, 17-1351-82, A2F10;CD34-A700, 56-0341-82, RAM34;Ki67-PE, 12-5698-82, SolA15<br>Antibodies (in the order of name, catalog number, and clone name) from BD Biosciences: streptavidin PE-Texas-Red , 551487; c-Kit/CD117-BV421, 562609, 2B8; c-Kit/CD117-PECy7, 558163, 2B8; CD45.1-A700, 561235, A20; Streptavidin PE-CF594 562284; Caspase3-PE, 550821;Biotinylated Hamster Anti-Mouse CD3e, 553060, 145-2C11; Biotinylated Rat Anti-Mouse CD4, 553045, RM4-5; Biotinylated Rat Anti-Mouse CD8a, 553029, 53-6.7; Biotinylated Rat Anti-Mouse Ter119, 553672, TER-119; Biotinylated Rat Anti-Mouse B220, 553086, RA3-6B2; Biotinylated Rat Anti-Mouse Gr-1,553124, RB6-8C5; Biotinylated Rat Anti-Mouse CD11b, 557395, M1/70;<br><br>Antibodies from Cell Signaling Technologies: HSP90 (Cell Signaling Technologies, 4874S).<br>α-H3K4me3 (Cell Signaling Technology, 9751),<br>Rabbit IgG (Cell Signaling Technology, 66362)<br>Antibodies from ENZO: HSF1 (ENZO ADI-SPA-901-F).<br>HSF1 (Invitrogen, Cat#MA5-27688) and/or CD34 (Dako, Cat#M7165)<br>Antibodies from Abcam: NDUFA9 (ab14713), SDHA (ab14715), SDHB (ab14714), SDHC (ab155999), SDHD (ab189945), UQCRC2 (ab14745), MTCO1 (ab14705), ATP5B (ab14730).<br>Antibodies from Origene: DDK/FLAG (Origene, TA180144)<br>Antibodies from Santa Cruz: beta-actin (Santa Cruz, sc-47778).
Validation	Validation of all antibodies was performed by the respective manufacturers they were purchased from. For the eBioscience and BD Bioscience antibodies used in Flow cytometry, detailed validation data can be found under the respective catalogue numbers on the manufacturers' websites. eBioscience: https://www.thermofisher.com/antibody/primary/query/filter/application/Flow%20Cytometry<br>BD Bioscience: https://www.bdbiosciences.com/en-us/products/reagents<br><br>For Antibodies from Cell Signaling Technologies, ENZO, Invitrogen, Dako, Abcam, Origene and Santa Cruz, detailed validation data can be found as follows:<br>HSP90 (https://www.cellsignal.com/products/primary-antibodies/hsp90-antibody/4874), α-H3K4me3(https://www.cellsignal.com/products/primary-antibodies/tri-methyl-histone-h3-lys4-c42d8-rabbit-mab/9751), Rabbit IgG (https://www.cellsignal.com/products/primary-antibodies/rabbit-da1e-mab-igg-xp-isotype-control-cut-amp-run/66362)<br>HSF1 antibody from ENZO (ADI-SPA-901-F): https://www.enzolifesciences.com/ADI-SPA-901/hsf1-polyclonal-antibody/<br>HSF1 (Invitrogen, Cat#MA5-27688): https://www.thermofisher.com/antibody/product/HSF1-Antibody-clone-10H8-Monoclonal/MA5-27688<br>CD34 (Dako, Cat#M7165): https://www.agilent.com/en/product/immunohistochemistry/antibodies-controls/primary-antibodies/cd34-class-ii-%28concentrate%29-76634<br>NDUFA9 (ab14713): https://www.abcam.com/ndufa9-antibody-20c11b11b11-ab14713.html<br>SDHA (ab14715): https://www.abcam.com/sdha-antibody-2e3gc12fb2ae2-ab14715.html<br>SDHB (ab14714): https://www.abcam.com/sdhb-antibody-21a11ae7-ab14714.html<br>SDHC (ab155999): https://www.abcam.com/sdhc-antibody-epr11035b-ab155999.html<br>SDHD (ab189945): https://www.abcam.com/sdhd-antibody-ab189945.html<br>UQCRC2 (ab14745): https://www.abcam.com/uqcrc2-antibody-13g12af12bb11-ab14745.html<br>MTCO1 (ab14705): https://www.abcam.com/mtco1-antibody-1d6e1a8-ab14705.html<br>ATP5B (ab14730): https://www.abcam.com/atpb-antibody-3d5-mitochondrial-marker-ab14730.html<br>DDK/FLAG (Origene, TA180144) : https://www.origene.com/catalog/antibodies/tag-antibodies/ta180144/ddk-flag-mouse-monoclonal-antibody-clone-oti11c3<br>Cruz: beta-actin (Santa Cruz, sc-47778): https://www.scbt.com/p/beta-actin-antibody-c4

Eukaryotic cell lines

Policy information about cell lines

Cell line source(s)	293T cell line for producing retroviruses was purchased from Takara/Clontech (authentication was performed by the supplier). The Human AML cell lines, NOMO-1 (ACC 542) purchased from Leibniz Institute DSMZ-German Collection of Microorganisms and Cell Cultures and MV4-11 (CRL-9591), were purchased from ATCC.
Authentication	Cell lines were obtained from verified sources and examined of morphology and growth characteristics.
Mycoplasma contamination	The cell lines used were negative for mycoplasma contamination.
Commonly misidentified lines<br>(See ICLAC register)	No commonly misidentified cell lines were used.

Animals and other organisms

Policy information about studies involving animals; ARRIVE guidelines recommended for reporting animal research

Laboratory animals	HSF1 fl/fl mice were kindly provided by Dr. Elisabeth Christians. The ROSACre-ERT2 mice were kindly provided by Dr. Yiying Zhang. Vav-Cre mice (Stock 008610) and the transplant recipient CD45.1 mice (stock 002014) and NSGS mice (stock# 013062) were purchased from Jackson Laboratory. All the mouse strains (except NSGS) are in a C57BL6 background and were used at 8–12 weeks old and included male and female mice.
Wild animals	No wild animals were used in this study.
Field-collected samples	Our study did not involve samples collected from the field.
Ethics oversight	The mouse experiments were conducted under approved protocol 2020-0031 of the Institutional Animal Care and Use Committee (IACUC) of Case Western Reserve University..

Note that full information on the approval of the study protocol must also be provided in the manuscript.

Human research participants

Policy information about studies involving human research participants

Population characteristics	De-identified human AML cells were obtained through the CRWU Hematopoietic Biorepository & Cellular Therapy Core (IRB STUDY20210216) and the flow cytometry laboratory at the University of Iowa (Institutional IRB Approval #201508729). Archived human AML tissue blocks was performed at the University of Iowa under protocol "The role of HSF1 in regulating hematopoietic and leukemic stem cells (IRB # 201903742)".
Recruitment	Patients were not recruited for the study
Ethics oversight	AML samples were obtained from through the CRWU Hematopoietic Biorepository & Cellular Therapy Core (IRB STUDY20210216), the flow cytometry laboratory at the University of Iowa (Institutional IRB Approval #201508729), and pathology archive at University of Iowa (IRB # 201903742).

Note that full information on the approval of the study protocol must also be provided in the manuscript.

Flow Cytometry

Plots

Confirm that:

- [x] The axis labels state the marker and fluorochrome used (e.g. CD4-FITC).
- [x] The axis scales are clearly visible. Include numbers along axes only for bottom left plot of group (a 'group' is an analysis of identical markers).
- [x] All plots are contour plots with outliers or pseudocolor plots.
- [x] A numerical value for number of cells or percentage (with statistics) is provided.

Methodology

Sample preparation	Bone marrow, spleen, peripheral blood and lymph nodes was collected from mice and red blood cells were lysed. For immunostaining, cells were passed through a 70 micron strainer, blocked for 15 minutes on ice with mouse Fc-blocking reagent (BD), and stained for 30 minutes on ice in PBS supplemented with 2% FBS.
Instrument	BD LSR II and Aria
Software	FlowJo (TreeStar).
Cell population abundance	For some experiments, purity was reached 99%.
Gating strategy	Samples for analysis, sorting into subpopulations for sequencing and adoptive transfer were gated on live cells based upon SSC-A and FSC-A, then gated on singlets based upon FSC-A and SSC-W, then gated on interested populations according to experiments and sorted as described in the manuscript.<br>Recipient mice analysis – Cells from peripheral blood, bone marrow, or spleen were gated on live cells based upon SSC-A and FSC-A, then gated on singlets based upon FSC-A and SSC-W, then gated on donor CD45.2 positive cells for analysis of engrafted immunophenotype.

- [x] Tick this box to confirm that a figure exemplifying the gating strategy is provided in the Supplementary Information.
