# Supplementary material for: HSF1 is a driver of leukemia stem cell self-renewal in acute myeloid leukemia
Source: Nat Commun. 2022 Oct 16;13:6107. doi: 10.1038/s41467-022-33861-1 (PMC9573868; doi:10.1038/s41467-022-33861-1)
Supplement: Supplementary file 4 — Source Data [file 41467_2022_33861_MOESM4_ESM.zip › 345917_2_data_set_6944047_rj0867.pptx]

## Slide 1
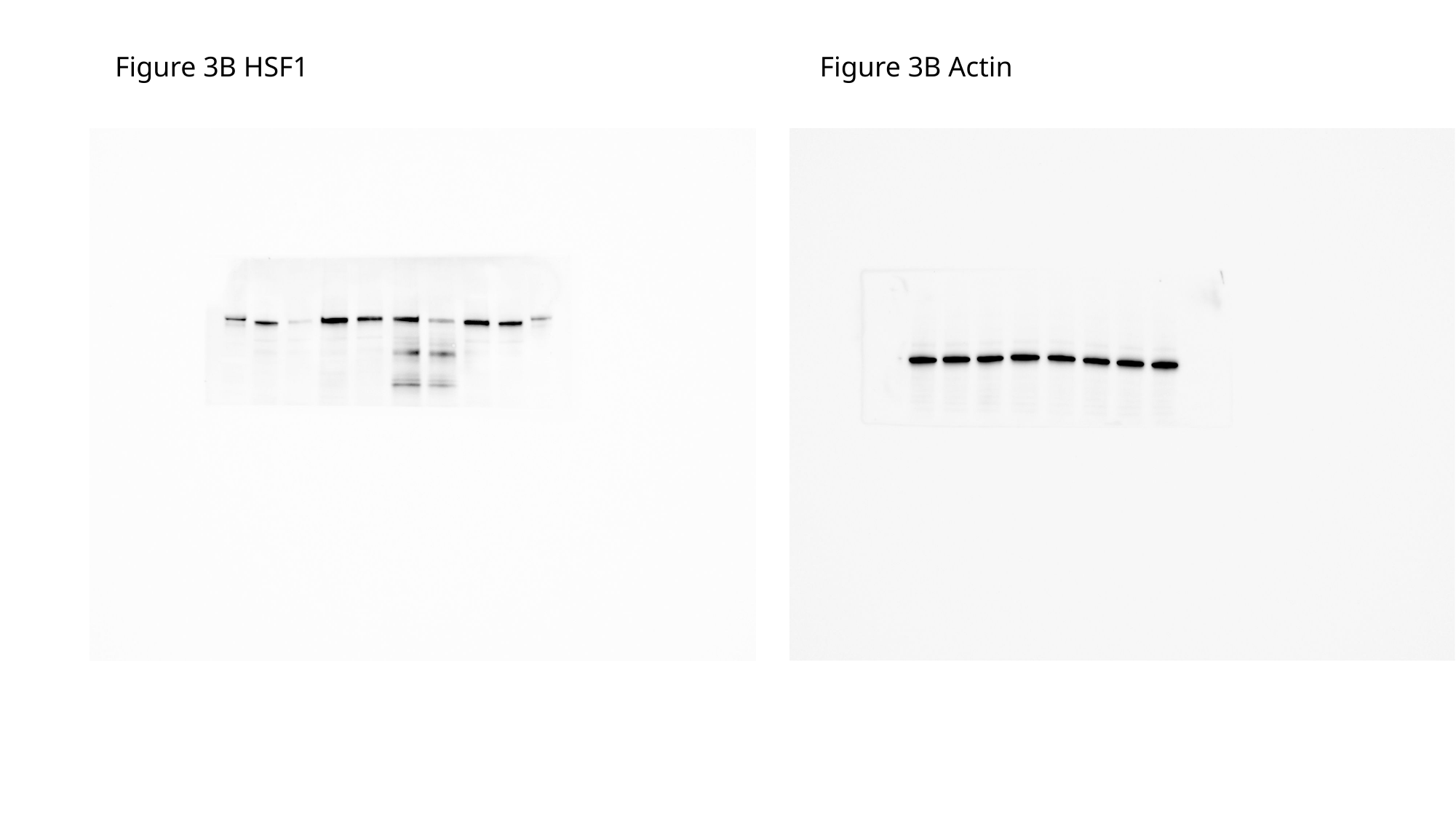

Figure 3B HSF1
Figure 3B Actin

## Slide 2
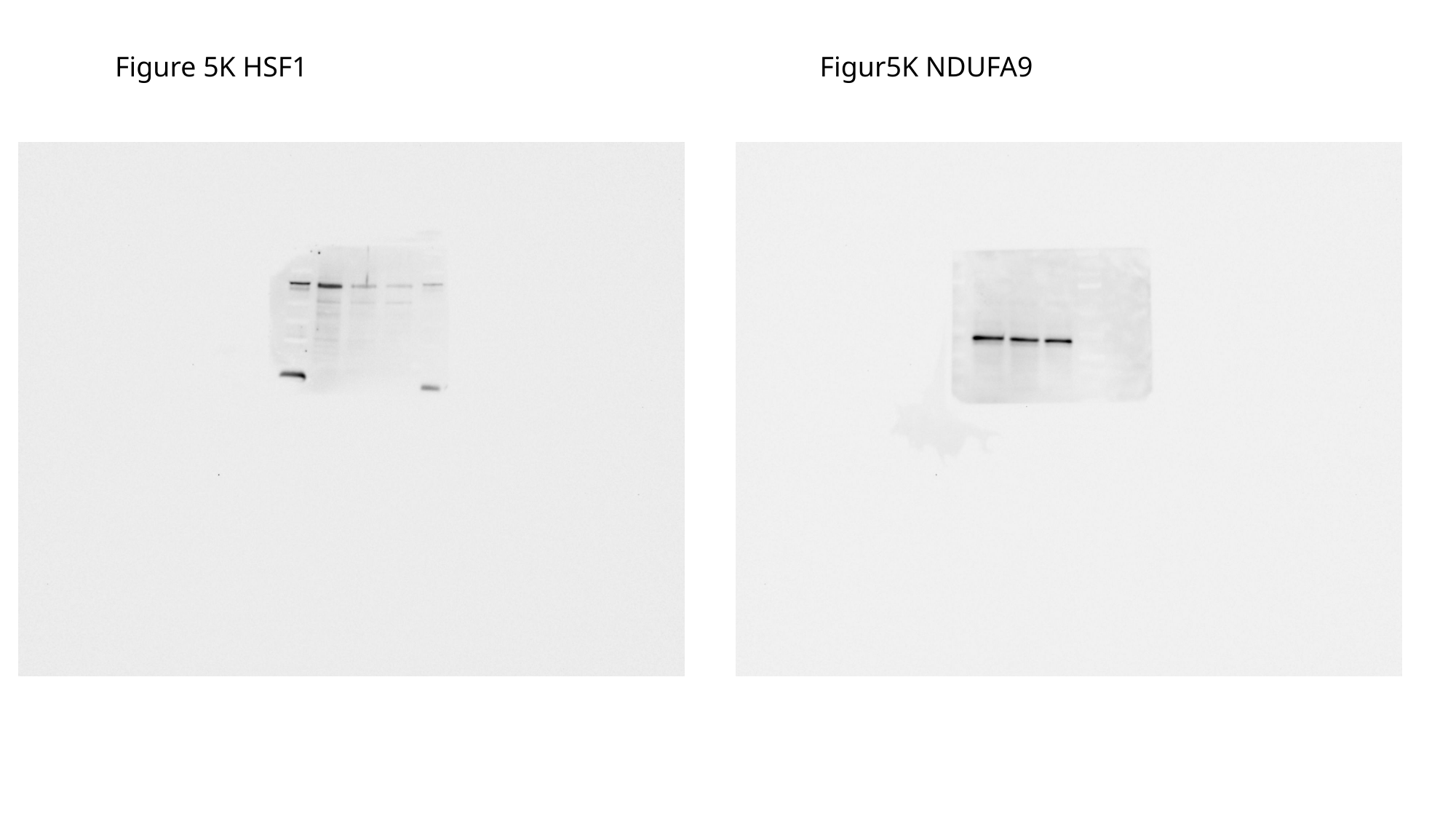

Figure 5K HSF1
Figur5K NDUFA9

## Slide 3
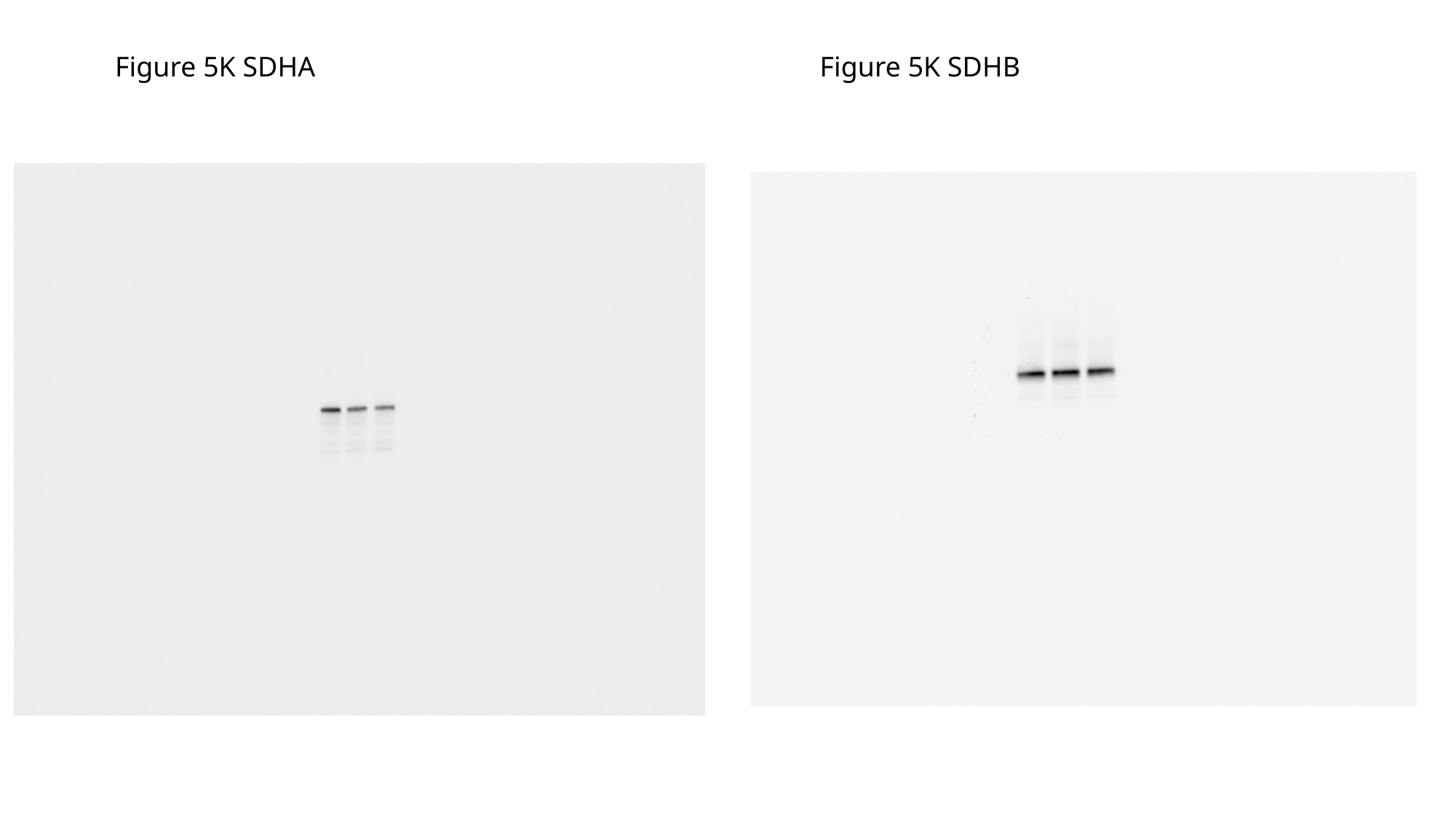

Figure 5K SDHA
Figure 5K SDHB

## Slide 4
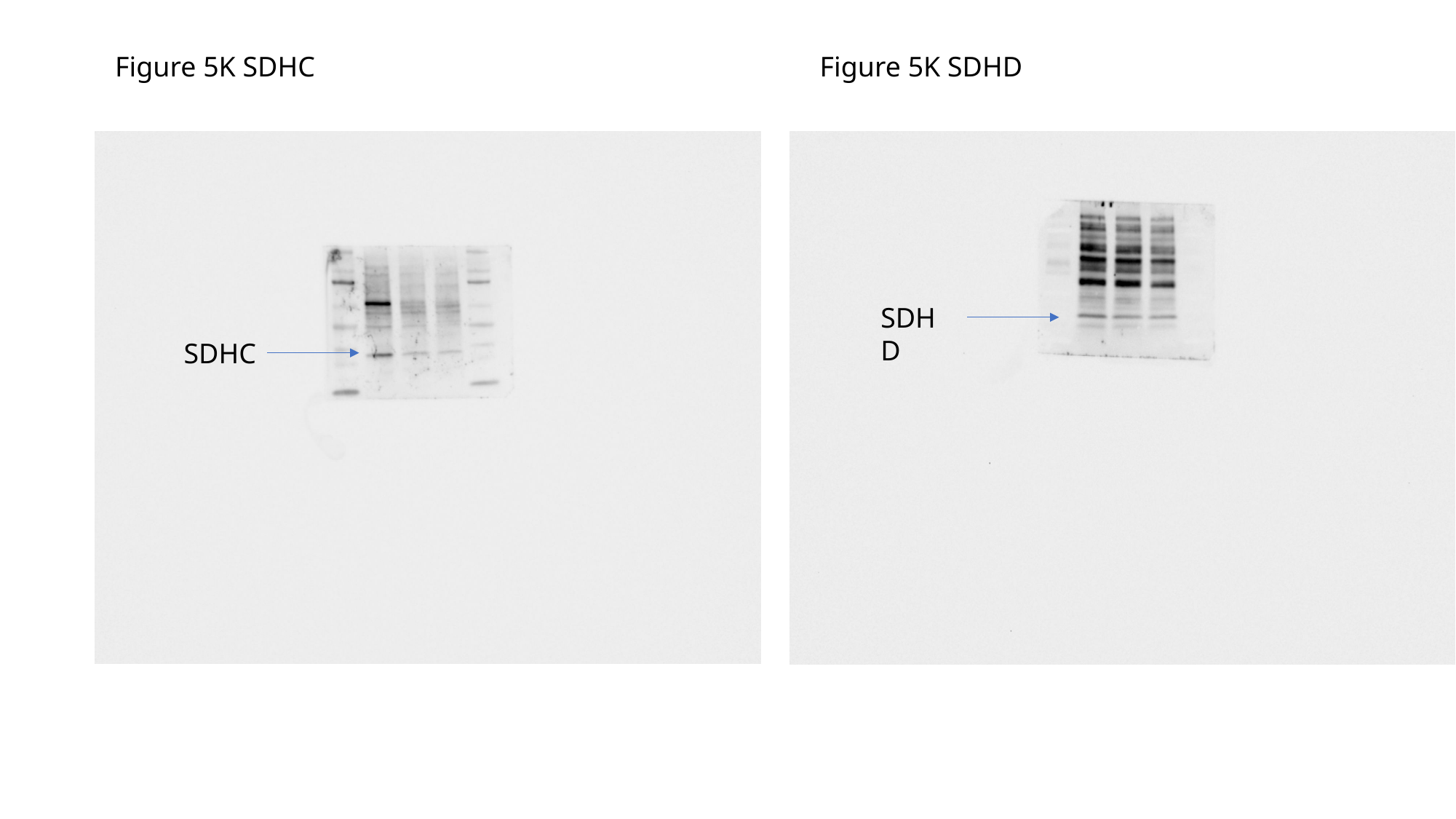

Figure 5K SDHC
Figure 5K SDHD
SDHD
SDHC

## Slide 5
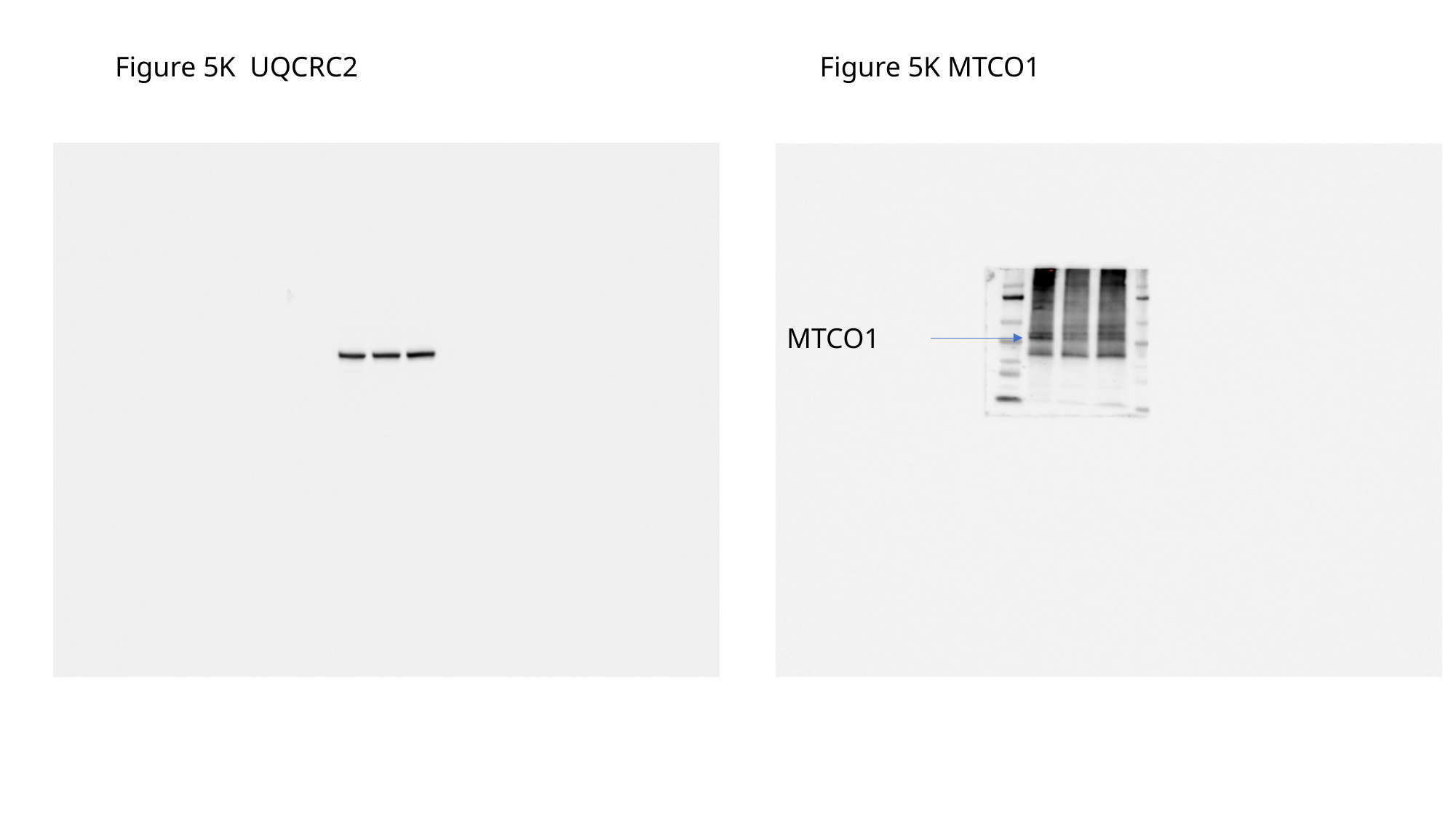

Figure 5K UQCRC2
Figure 5K MTCO1
MTCO1

## Slide 6
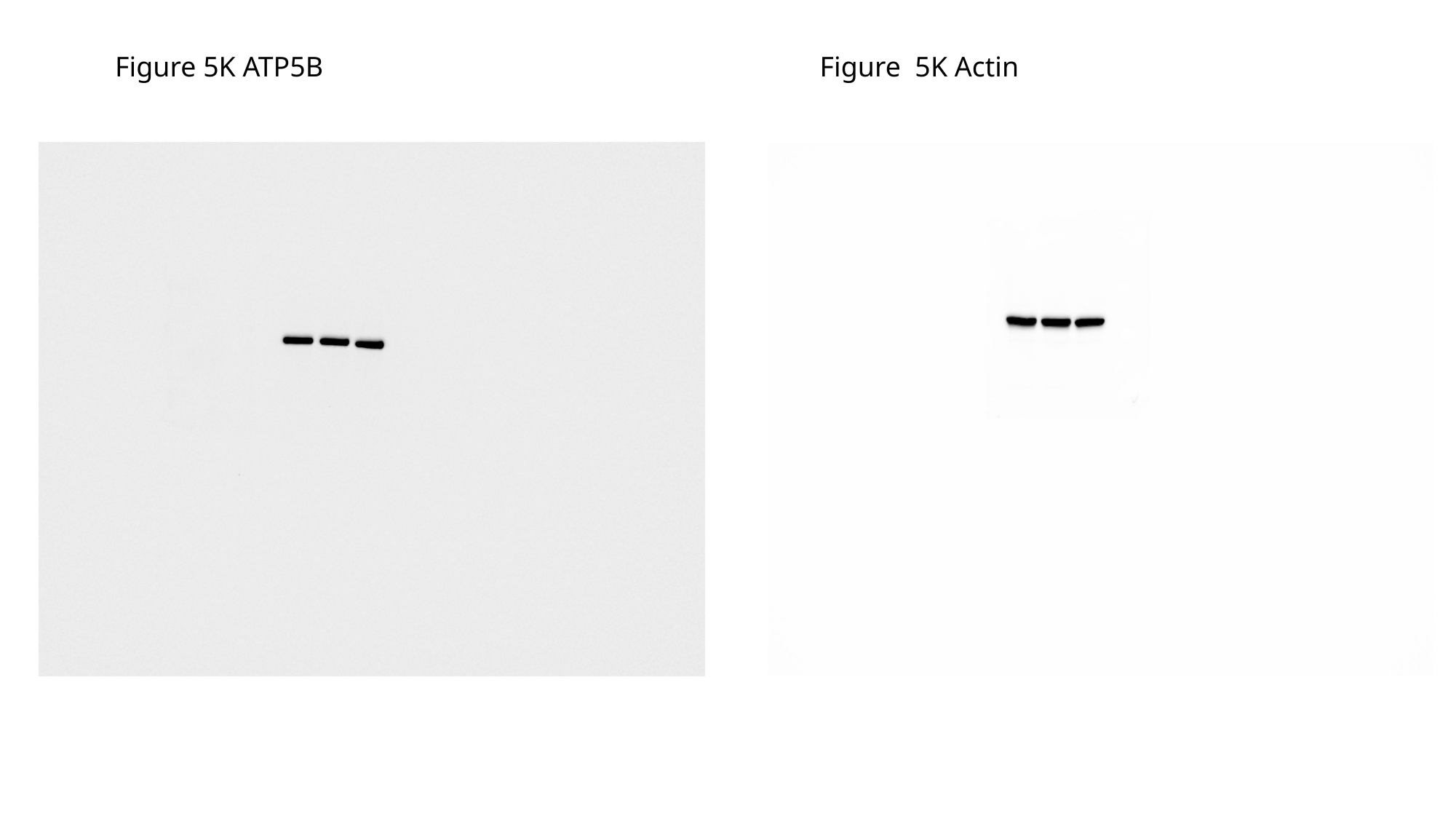

Figure 5K ATP5B
Figure 5K Actin

## Slide 7
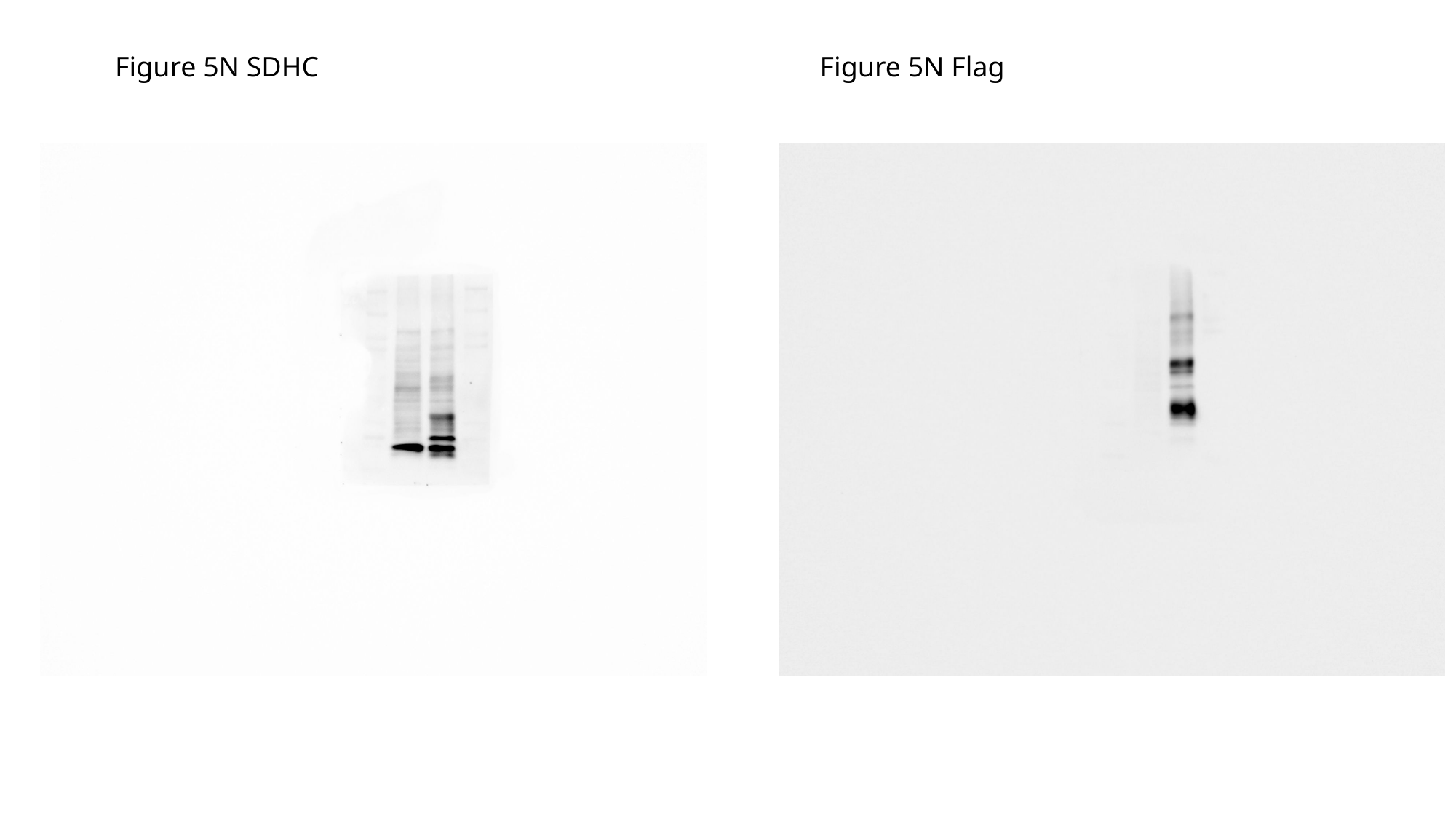

Figure 5N SDHC
Figure 5N Flag

## Slide 8
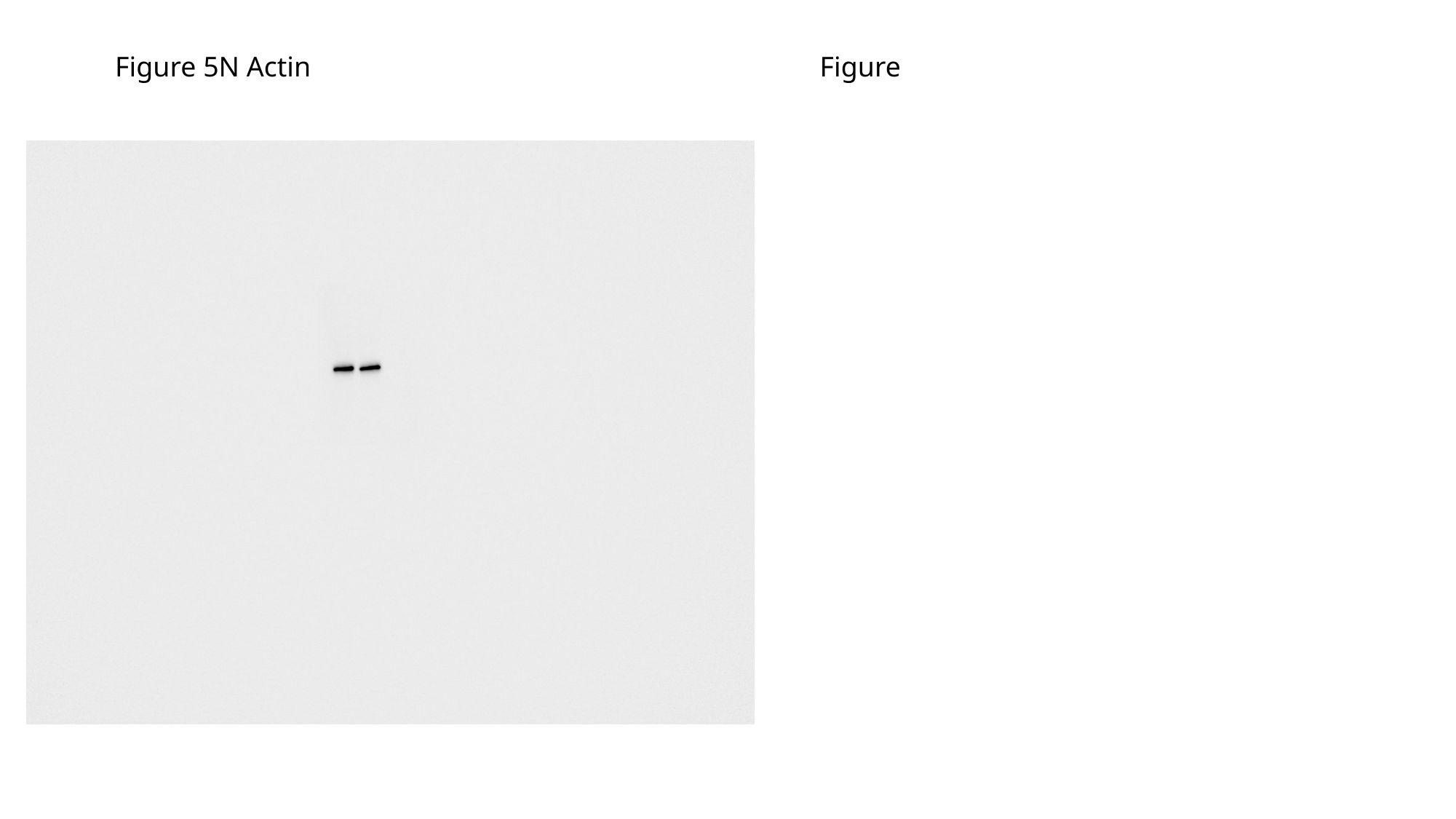

Figure 5N Actin
Figure

## Slide 9
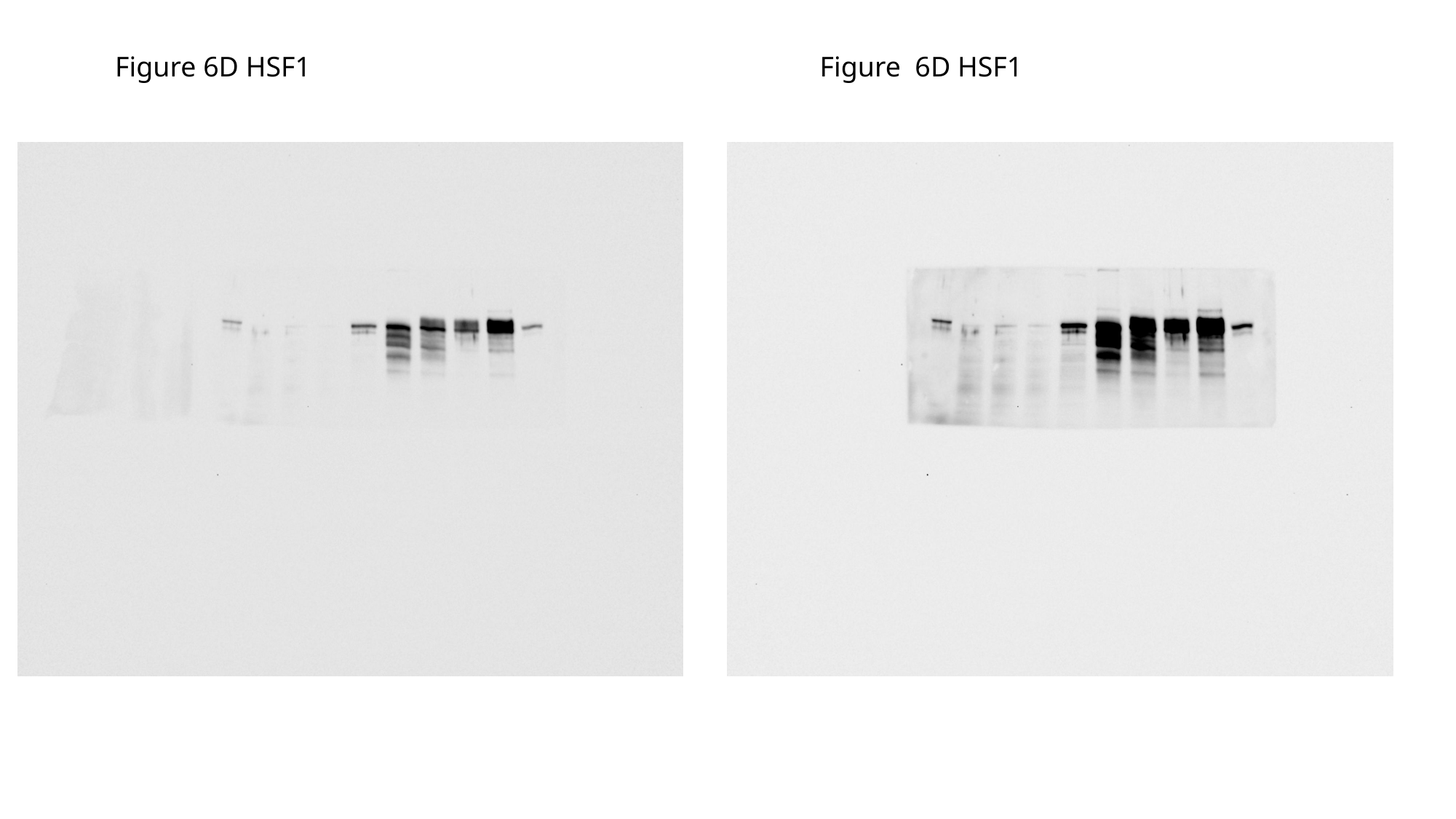

Figure 6D HSF1
Figure 6D HSF1

## Slide 10
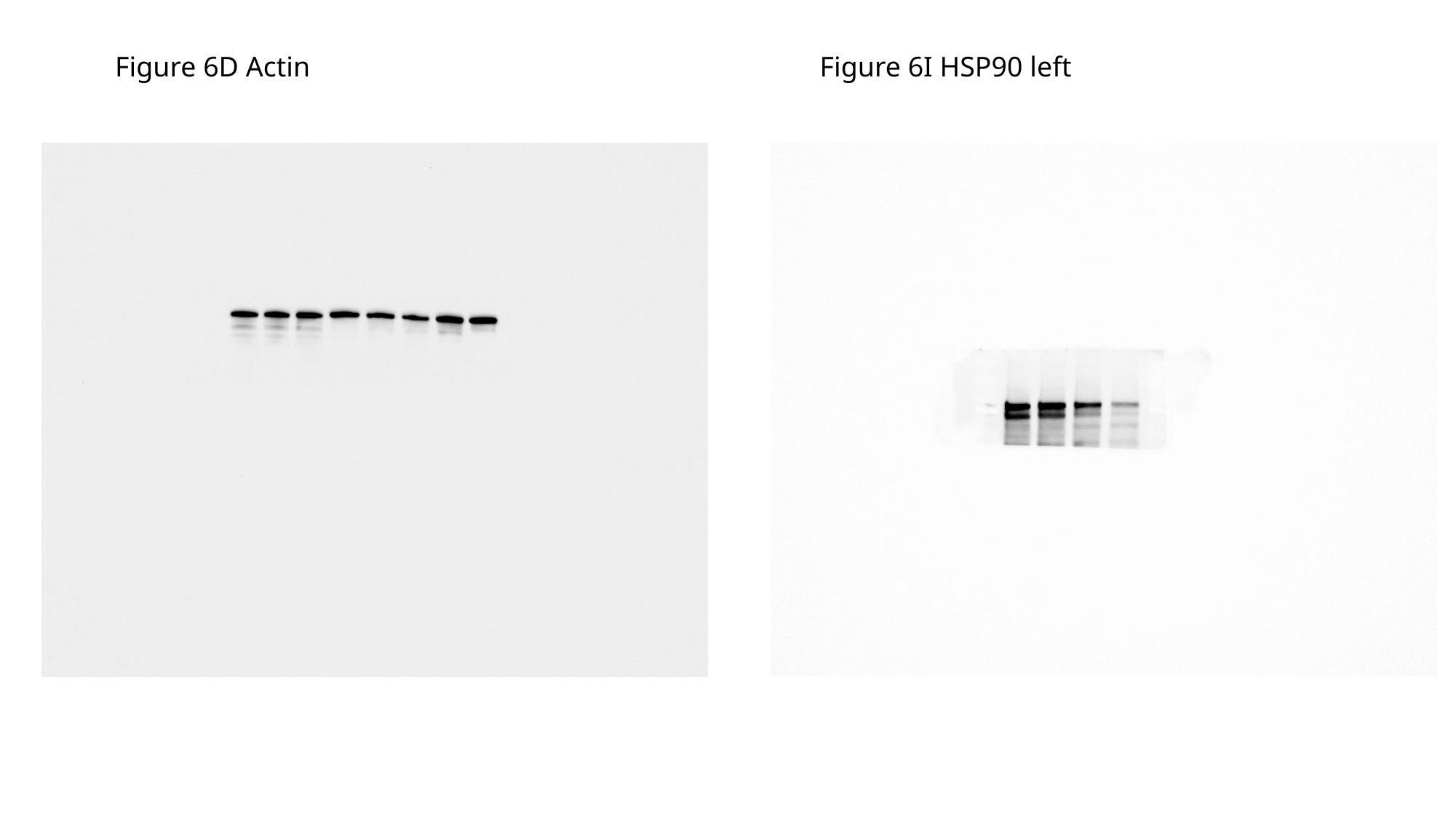

Figure 6D Actin
Figure 6I HSP90 left

## Slide 11
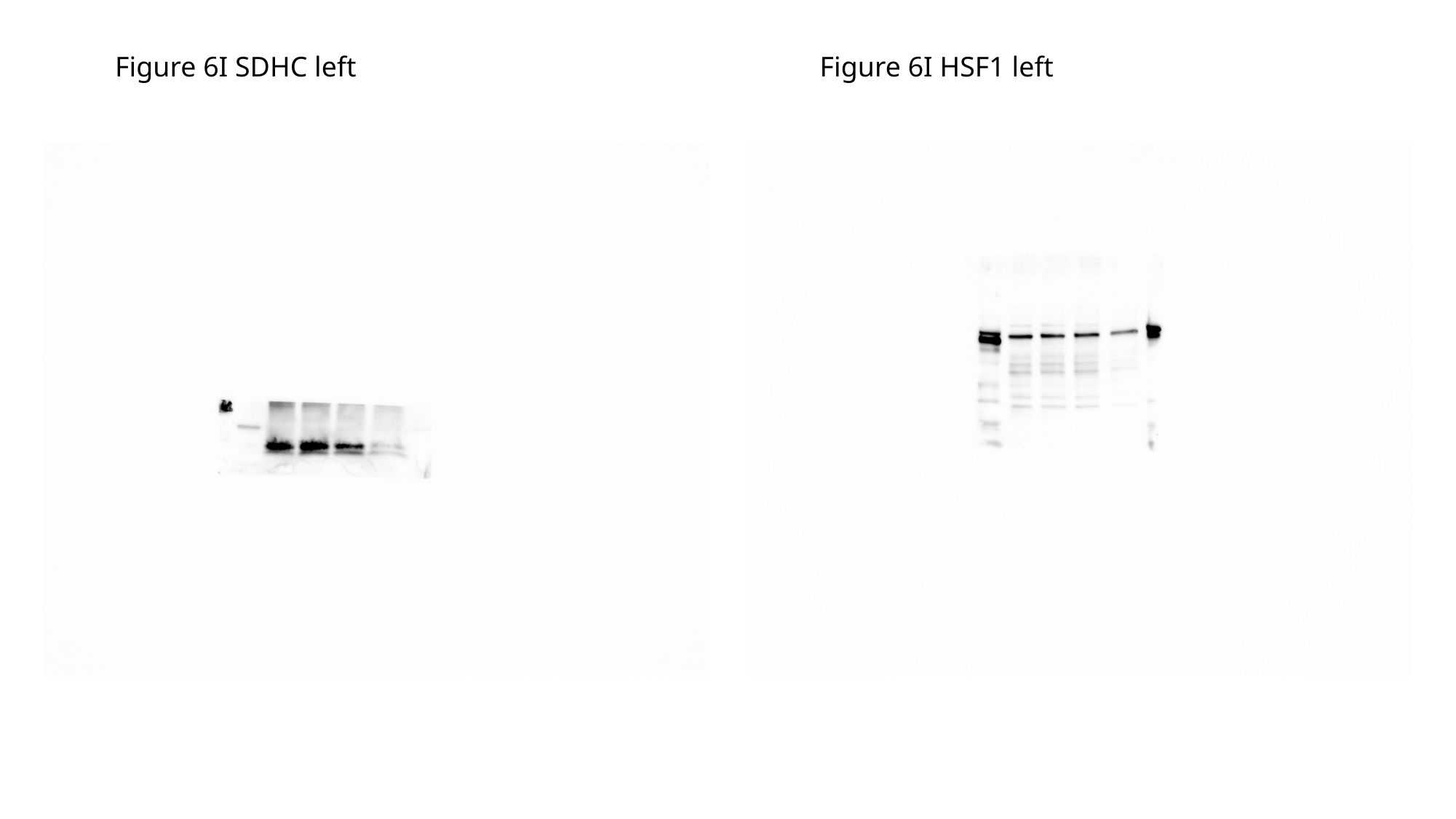

Figure 6I SDHC left
Figure 6I HSF1 left

## Slide 12
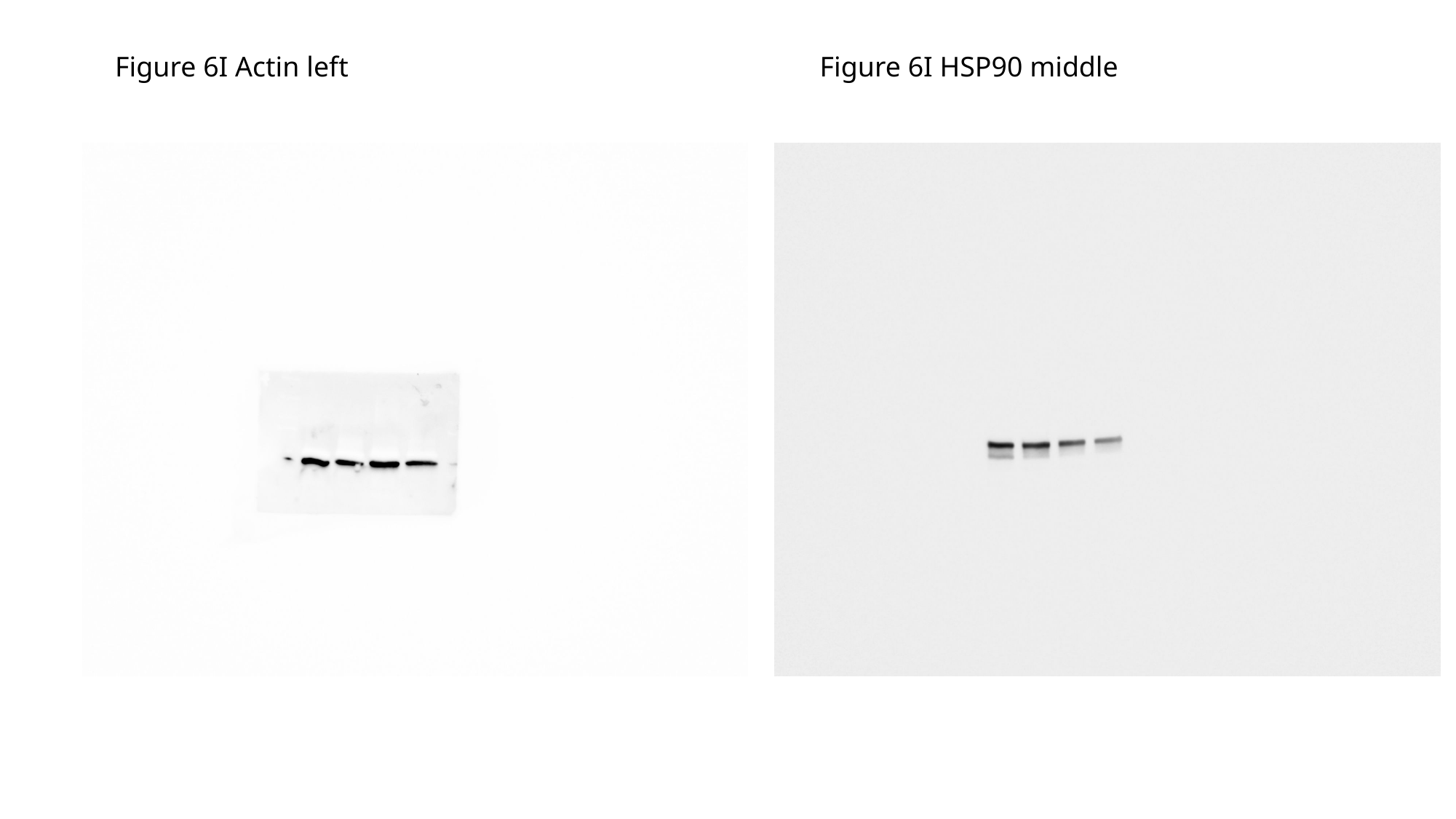

Figure 6I Actin left
Figure 6I HSP90 middle

## Slide 13
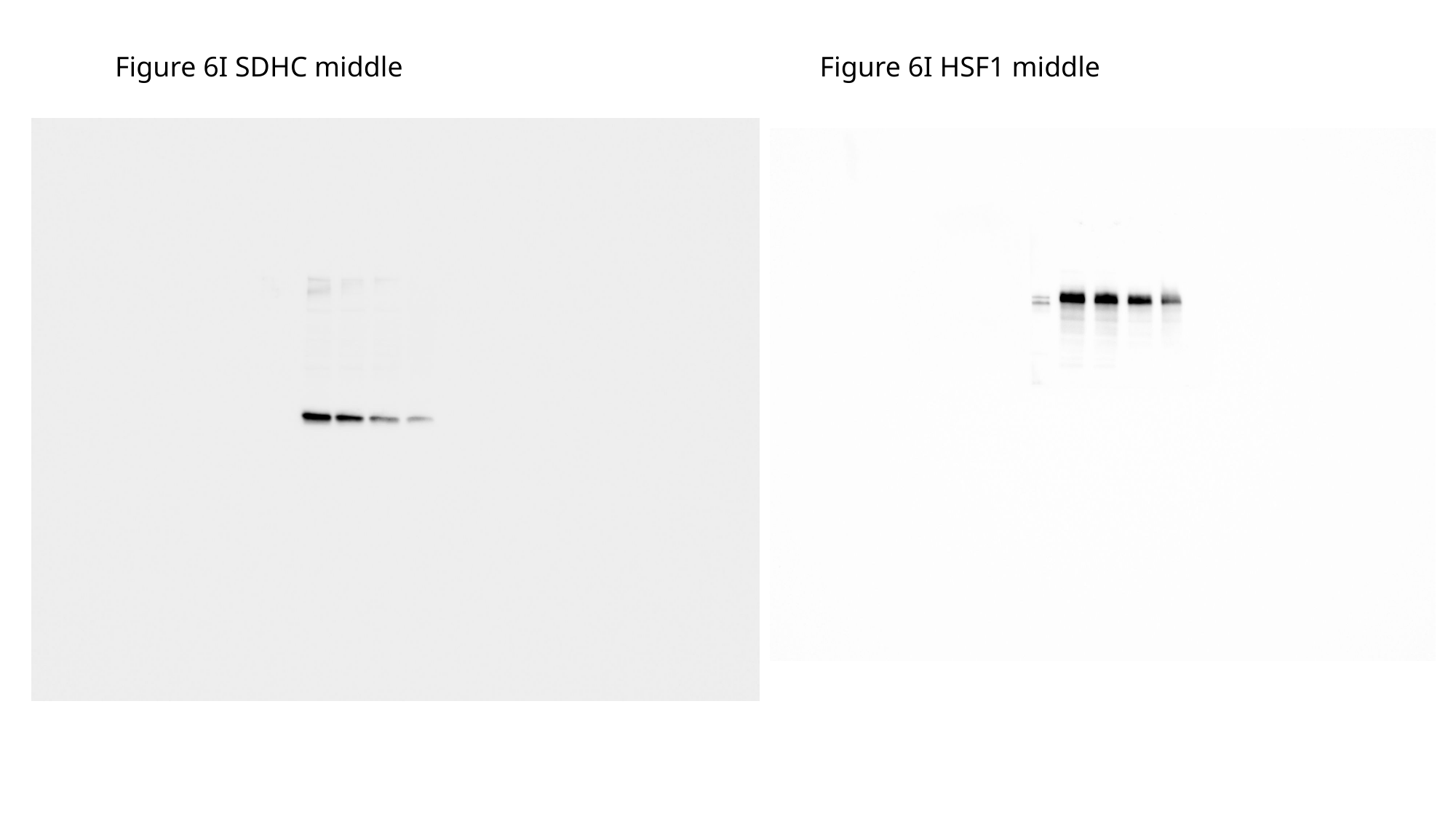

Figure 6I SDHC middle
Figure 6I HSF1 middle

## Slide 14
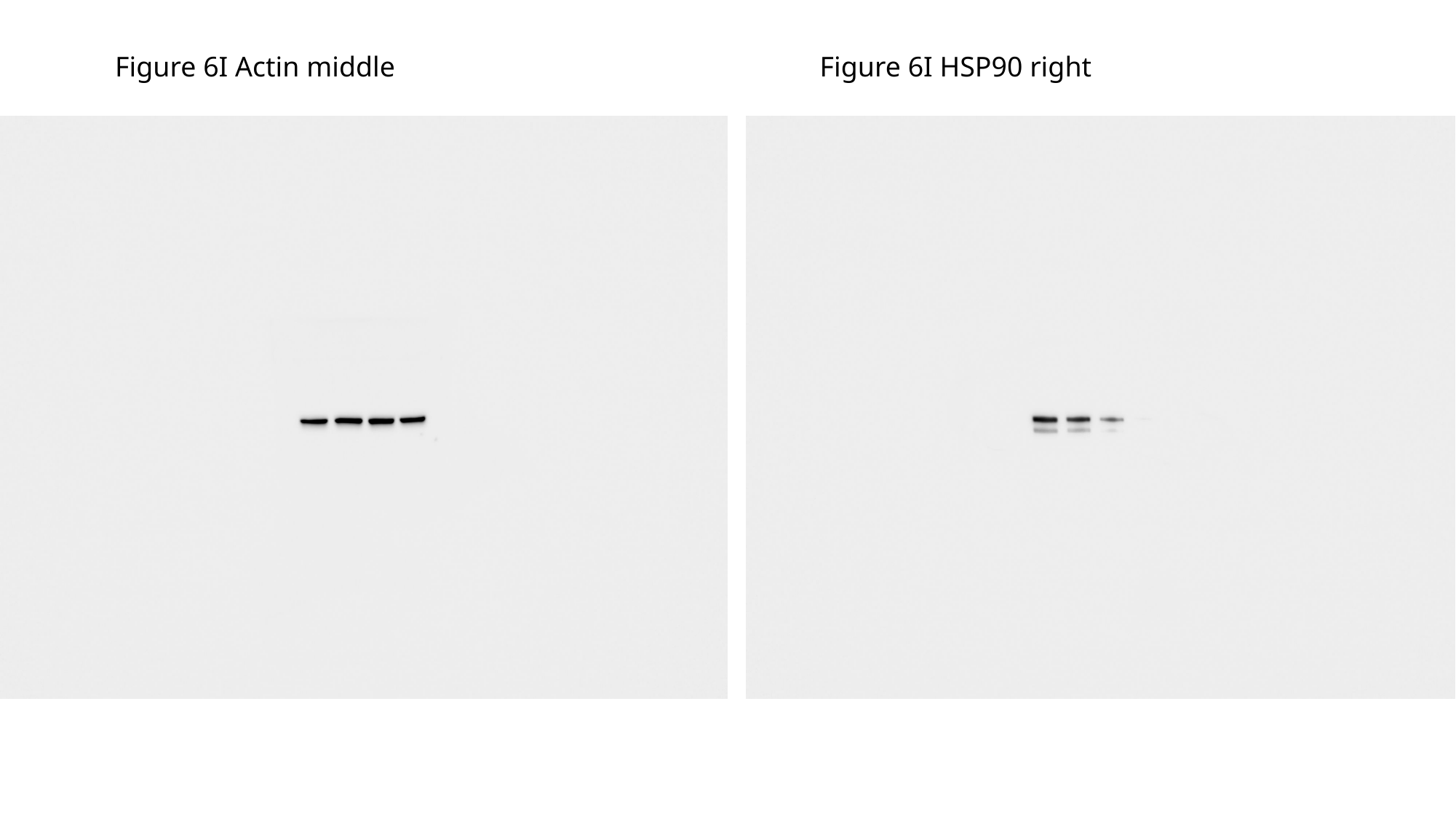

Figure 6I Actin middle
Figure 6I HSP90 right

## Slide 15
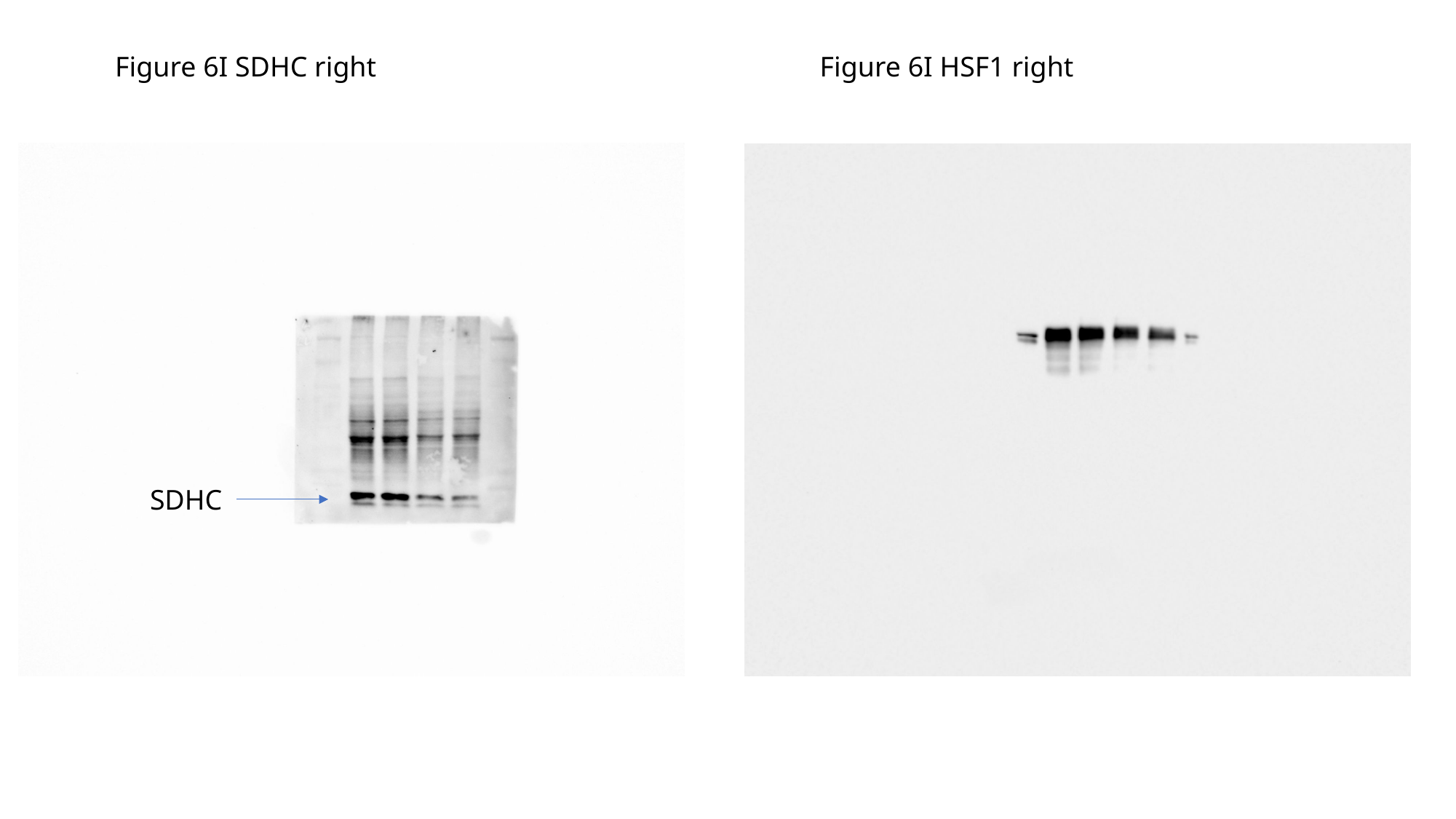

Figure 6I SDHC right
Figure 6I HSF1 right
SDHC

## Slide 16
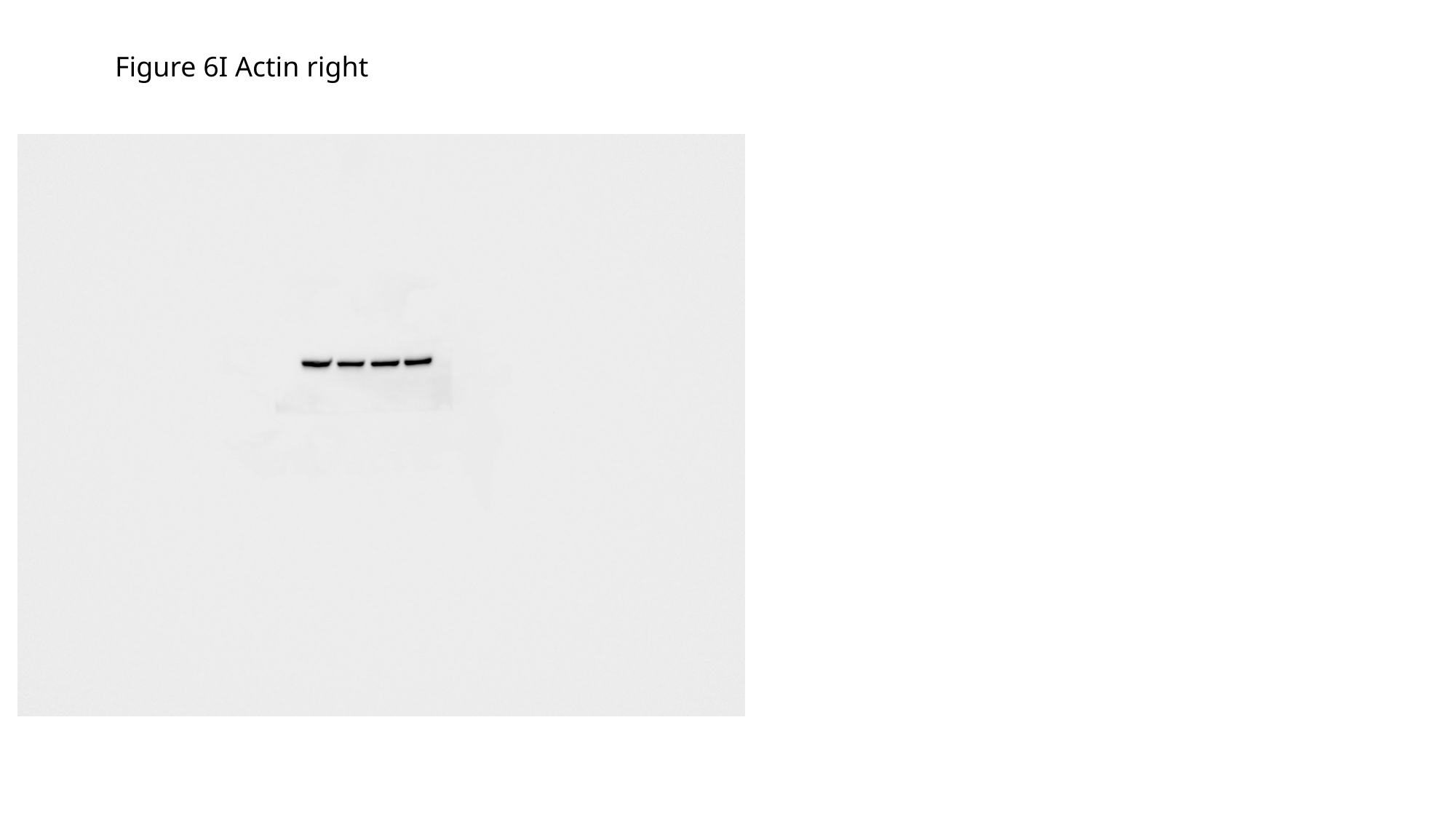

Figure 6I Actin right

## Slide 17
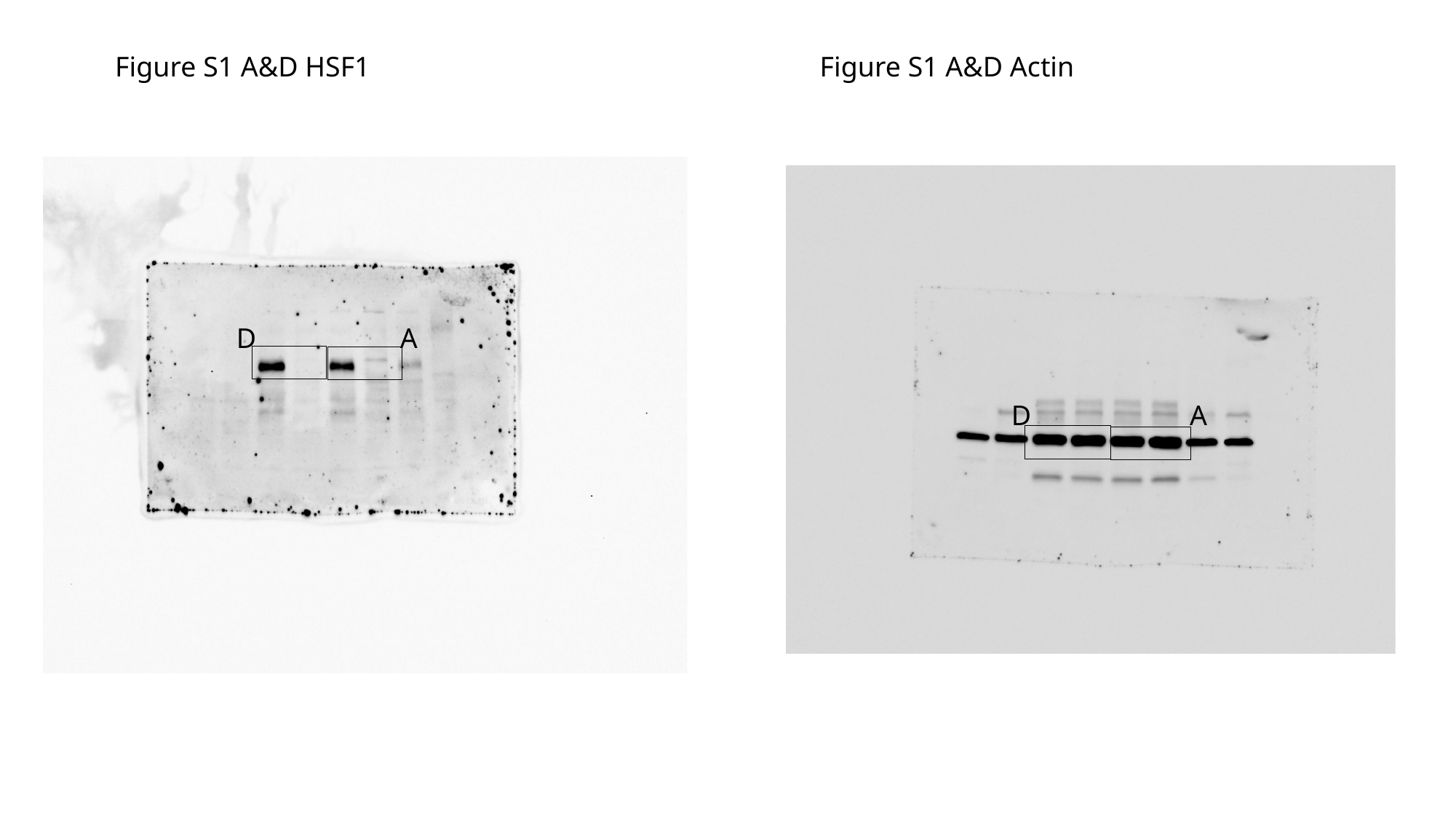

Figure S1 A&D HSF1
Figure S1 A&D Actin
D
A
D
A

## Slide 18
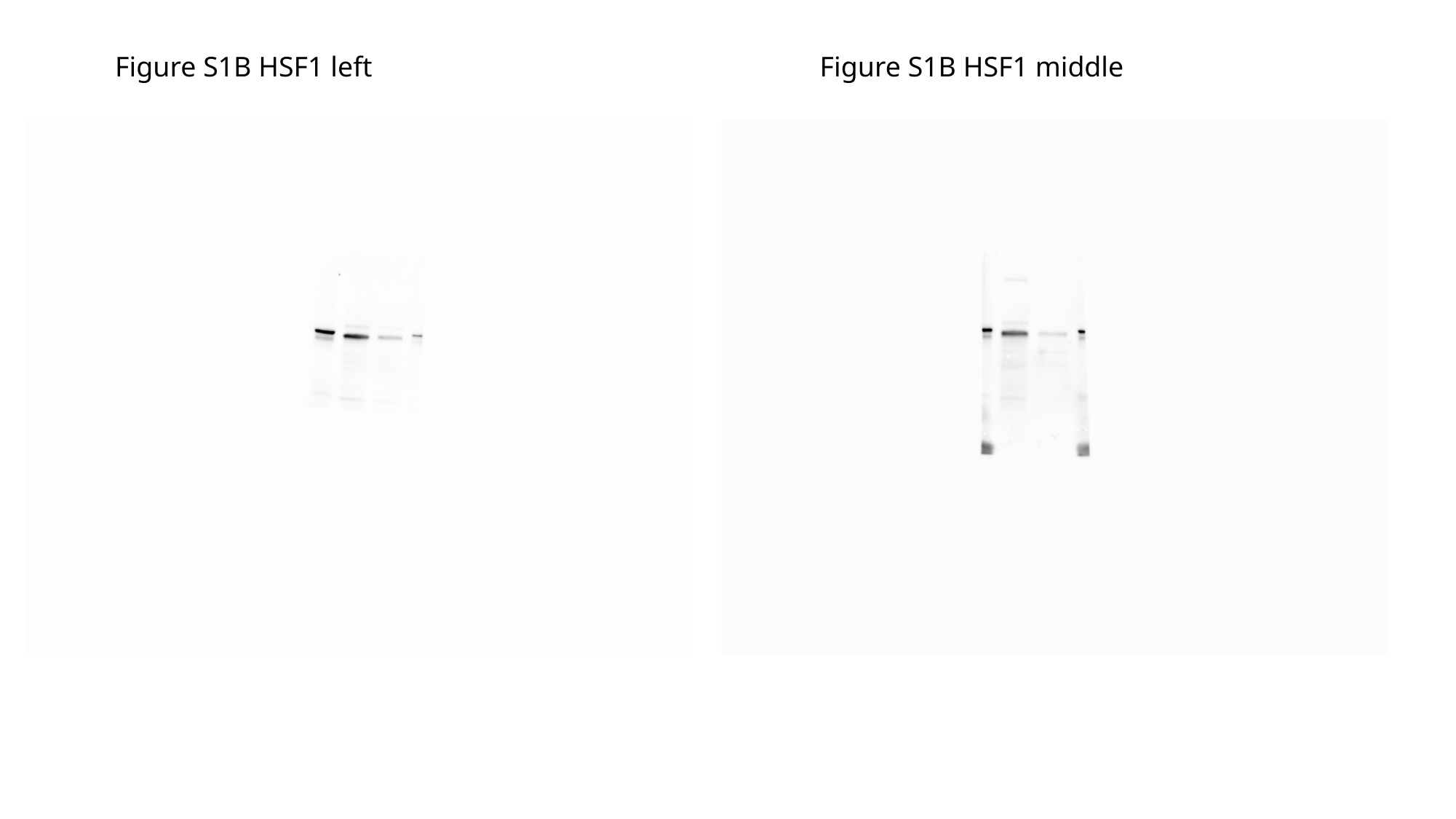

Figure S1B HSF1 left
Figure S1B HSF1 middle

## Slide 19
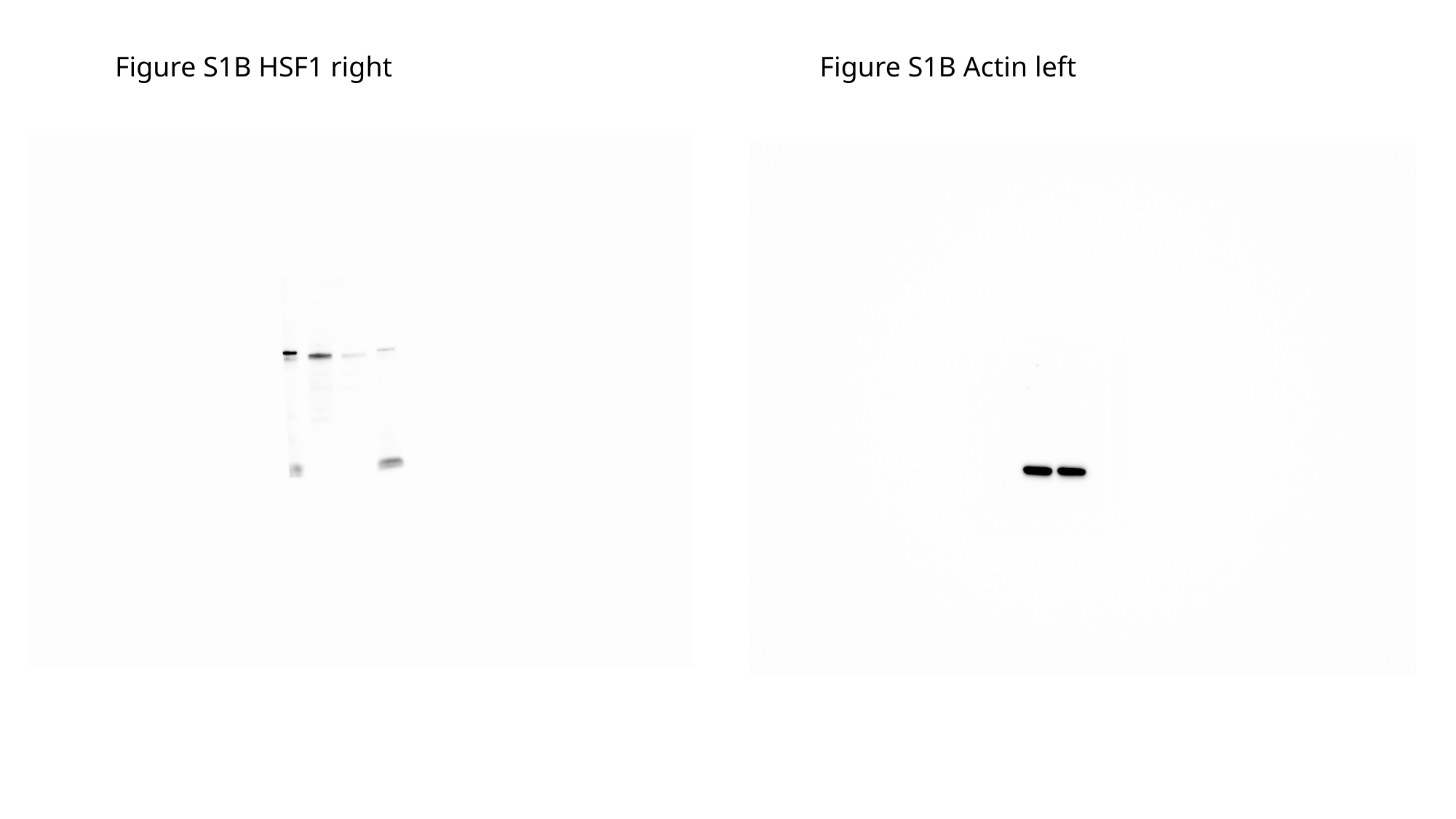

Figure S1B HSF1 right
Figure S1B Actin left

## Slide 20
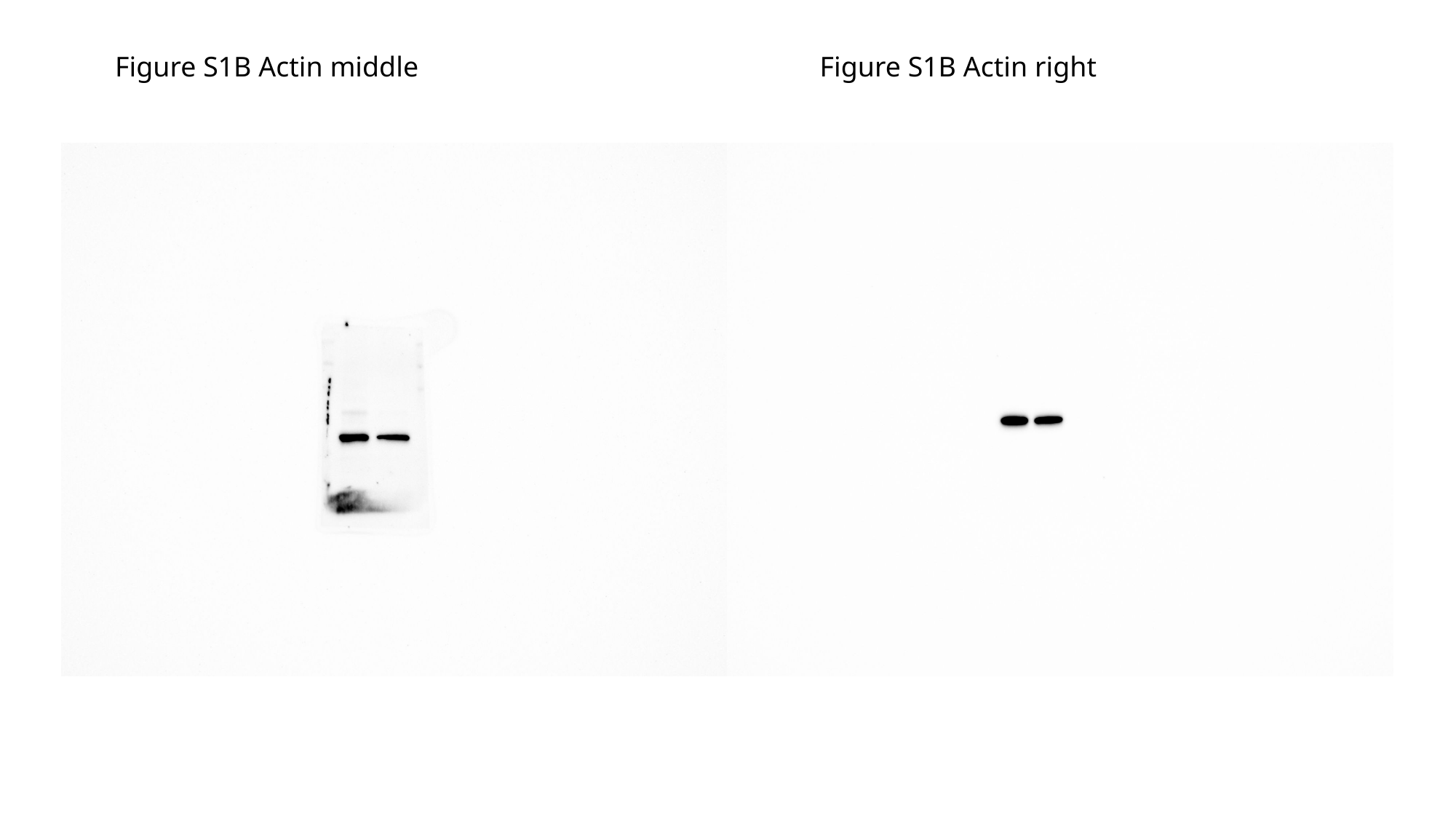

Figure S1B Actin middle
Figure S1B Actin right

## Slide 21
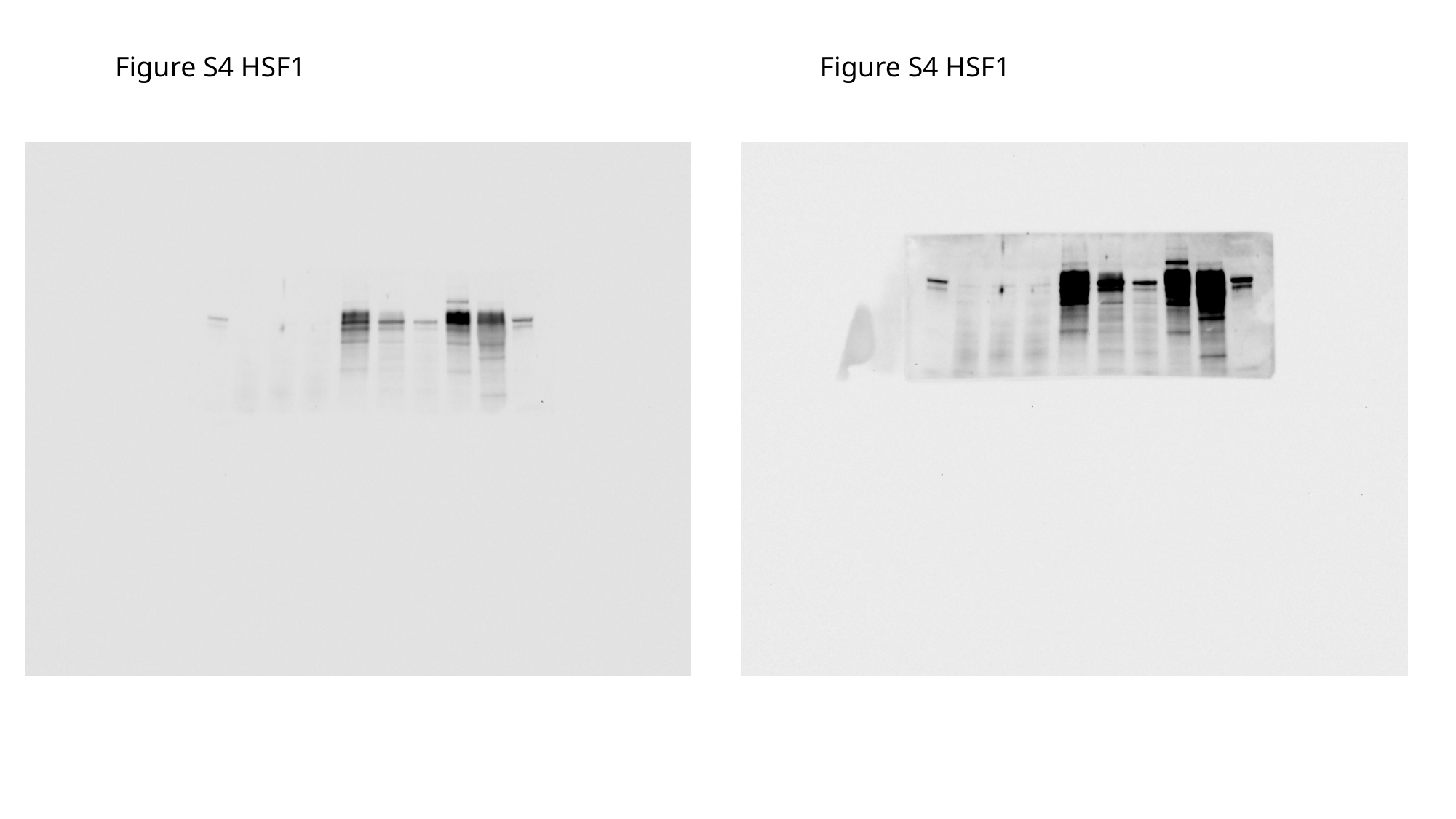

Figure S4 HSF1
Figure S4 HSF1

## Slide 22
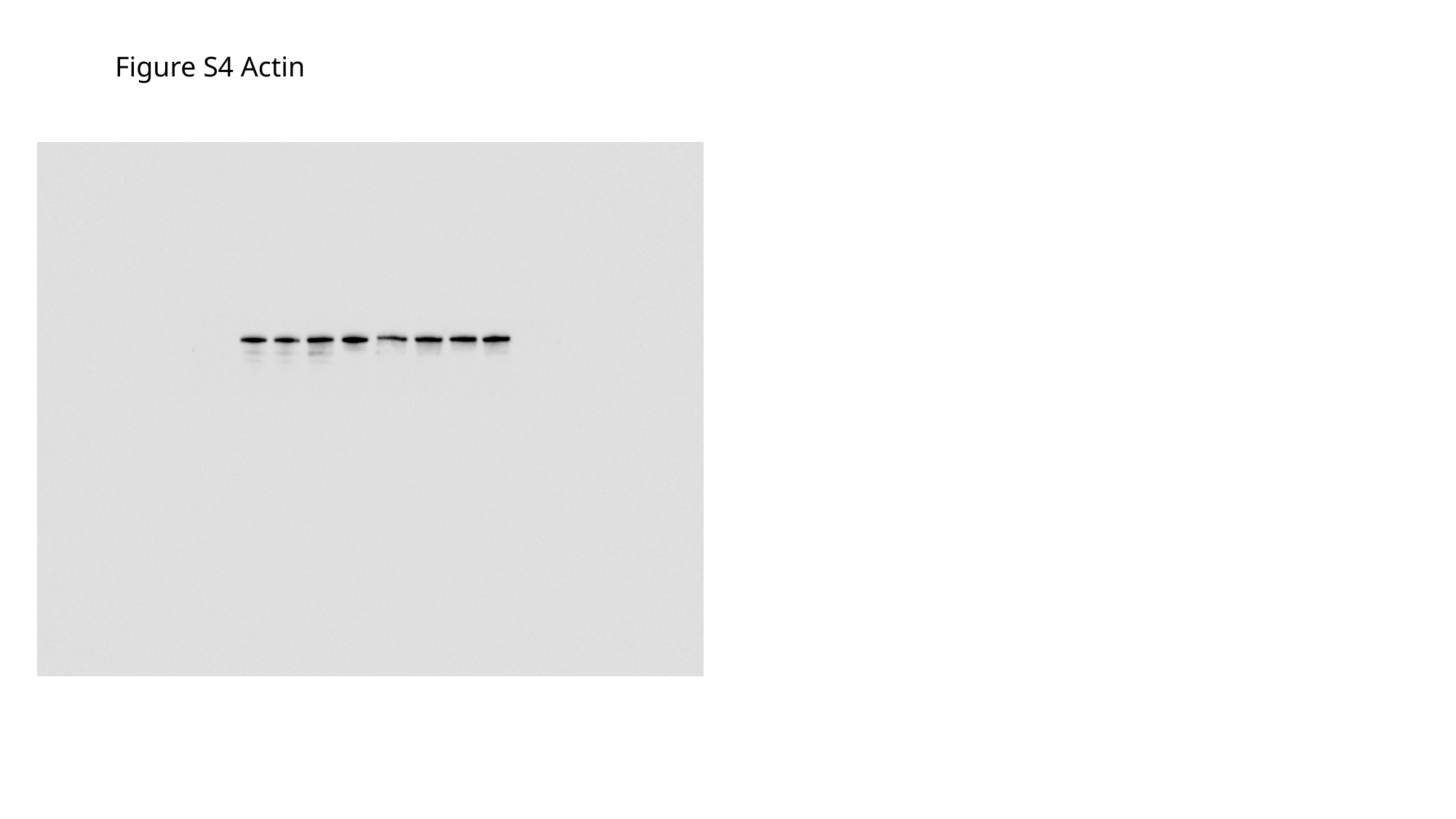

Figure S4 Actin
